# The price of survival: comparative adaptation to high altitudes between yaks and cattle

**DOI:** 10.1186/s42523-026-00584-3

**Published:** 2026-06-06

**Authors:** Lang Tan, Xiaojing Liu, Yonggui Ma, Jinfen Yang, Qunying Zhang, Jianbo Zhang, Binqiang Bai, Heng Ma, Ru Meng, Allan Degen, Nikola Palevich, Peijun Shi, Lizhuang Hao

**Affiliations:** 1 Key Laboratory of Plateau Grazing Animal Nutrition and Feed Science of Qinghai Province, Qinghai University, Xining, 810016 China; 2 State Key Laboratory of Animal Nutrition and Feeding, Beijing Engineering Technology Research Center of Raw Milk Quality and Safety Control, College of Animal Science and Technology, China Agricultural University, Beijing, 100193 China; 3 Academy of Plateau Science and Sustainability, Qinghai Normal University, Xining, 810008 China; 4 National Institute of Natural Hazards, Ministry of Emergency Management of China, Beijing, 100085 China; 5 Xining Animal Disease Prevention and Control Center, Xining, 810016 China; 6 Desert Animal Adaptations and Husbandry, Wyler Department of Dryland Agriculture, Blaustein Institutes for Desert Research, Ben-Gurion University of the Negev, Beer Sheva, 8410500 Israel; 7 AgResearch Group, Bioeconomy Science Institute, Grasslands Research Centre, Palmerston North, 4442 New Zealand; 8 State Key Laboratory of Earth Surface Processes and Disaster Risk Reduction, Faculty of Geographical Science, Beijing Normal University, Beijing, 100875 China; 9 Key Laboratory of Environmental Change and Natural Disaster, MOE, Beijing Normal University, Beijing, 100875 China

**Keywords:** High-altitude environments, Adaptation, Metabolism, Hypoxia, Yak

## Abstract

The yak (*Bos grunniens*) serves as an exceptional model for studying high-altitude adaptation mechanisms due to its evolutionary success in the hypoxic environment of the Qinghai-Tibet Plateau. Previous research has largely focused on genetic and physiological traits of yaks; however, the interactions between rumen microbiota and host physiology under hypoxic conditions are poorly understood. As the largest digestive organ in ruminants, the rumen and its microbiota play a central role in digestion and host nutrition. In this study, a comparative analysis of digestive metabolism and rumen microbiota was carried out in yaks and cattle (*Bos taurus*) under two distinct atmospheric oxygen scenarios: baseline (2,200 m) and hypoxic (3,800 m). Our findings reveal that yaks have developed unique microbial strategies to cope with energy deficits in hypoxic stress. These strategies include a shift in rumen microbiota toward amino acid degradation, providing more available energy substrates for host utilization, and enhanced long-chain fatty acid biosynthesis, enabling more efficient energy storage and utilization. This improves energy acquisition in yaks despite their reduced nutritional intake. However, this metabolic adaptation comes at a physiological cost - reduced microbial crude protein (MCP) synthesis, leading to elevated ruminal NH_3_-N levels, and increased fatty acid metabolism and urea cycle activity contributing to hepatic stress. Our results showed that under high-altitude conditions, yak MCP synthesis decreased by 47.3%; and ruminal NH3-N and serum ALT (a hepatic stress marker) increased by 147.2 and 19.7%, respectively. This study presents evidence of potential metabolic trade-offs in high-altitude adaptation, indicating that yaks may optimize microbially mediated energy production at the cost of liver health. These insights deepen our understanding of host-microbiome coevolution mechanisms in extreme environments and highlight biological costs associated with adaptation to high altitudes.

**Supplementary Information:**

The online version contains supplementary material available at 10.1186/s42523-026-00584-3.

## Background

The Qinghai-Tibet Plateau is known as the “Roof of the World” comprising the highest-altitude regions on Earth, with an average elevation of 3,000 m [1]. The most prominent feature of this area is lower atmospheric oxygen concentration (19.9 to 20.8%) compared to plains (21.0%) [2]. Hypoxia adversely affects the health of animals and can cause death in severe cases [3]. In studies involving humans and animal models, it has been demonstrated that exposure to chronic hypoxia can cause abnormal bone tissue structure, reduced bone density, stunted growth and development, and cardiovascular dysfunction [4, 5]. Epidemiological data from human populations indicate that the incidence of congenital cardiovascular diseases is much higher in high-altitude regions than in plains [6, 7]. This phenomenon, termed high-altitude hypoxic injury, has been reported in humans and non-ruminants but has not been documented in ruminants [8, 9].

The rumen, a unique digestive and metabolic chamber in ruminants, hosts a complex microbial community dominated by bacteria, which constitutes over 70% of its population. Although rumen is a strictly anaerobic environment, oxygen can still enter through feed and water intake, and influence microbial community structure even under oxygen-limited conditions [10]. Oxygen is essential for cellular respiration and various biochemical processes, it can also influence microbial structure in oxygen-limited environments [11]. Under hypoxic conditions, microbial communities exhibit altitude-dependent structural characteristics due to differences in oxygen sensitivity [12, 13]. In some studies [14], ruminal microbial communities differ significantly at high altitudes than those at lower altitudes. As a highly symbiotic ecosystem, the rumen microbiome forms an intricate metabolic network with the host [15, 16], and plays a critical role in nutrient acquisition [17], immune regulation and disease resistance [18–21]. However, systematic research exploring the microbe-host axis under extreme environments remains limited, particularly quantifying the functional plasticity and compensatory potential of microbial communities.

Earlier studies have focused mostly on the genetic and physiological adaptation mechanisms of yaks [22, 23], and modern reproductive technologies have also been explored to improve their production efficiency [24]. However, the role of rumen microbiota in this process remains poorly understood. To further investigate the role of rumen microbiodata, we used cattle as an ecological control in a two-phase experimental design (baseline: 2,200 m; hypoxia exposure: 3,800 m). Serum biochemical indicators, metabolomic profiles, rumen fermentation parameters, and microbial dynamics were monitored and compared between yaks and cattle. We hypothesized that yaks partially rely on rumen microbes to adapt to the hypoxic environment of the Qinghai-Tibet Plateau. Comparative analyses of serum biochemistry, serum metabolism, rumen fermentation parameters, and rumen microbiota were conducted to assess health-related adaptation between yaks and cattle. These findings aim to provide new theoretical insights into the health regulation mechanisms of high-altitude resilience in ruminants under hypoxic stress. Preliminary statistical analysis showed that, the slaughter rate and meat yield of local yaks decreased significantly with decreasing air oxygen levels. It suggests that traditional genetic adaptation theories alone may not fully explain the survival mechanisms of yaks at high altitudes, further emphasizing the need of this study.

## Methods

### Environmental factor measurement

Ror measuring temperature and humidity, an electronic meter (model: L92-1, Hangzhou LoggerTech Co., Ltd., China) was suspended in the center of the cowshed at a height of 1.5 m from the ground. Atmospheric pressure was measured at the two experimental sites. It was 77.3 and 63.8 kPa at 2,200 and 3,800 m altitude, respectively. The corresponding partial pressures of oxygen at these sites were 15.8 and 12.8 kPa, respectivly. Temperatures were recorded daily at 08:00, 12:00, and 18:00 during the last 7 days of the two-month experimental period, and their average were taken as daily temperature. A portable meter (model: MS104K-L, Qingdao Xinye Environmental Protection Technology Co., Ltd., China) was suspended adjacent to the temperature and humidity meter for measuring oxygen. The temperature, pH, and dissolved oxygen (DO) of drinking water for the animals were measured using a multi-parameter analyzer following the manufacturer’s instructions.

### Meat production and slaughter rate

Meat production per yak (kg) was calculated by dividing the total meat production of yaks by the number of yaks slaughtered in the same year. Slaughter rate (%) of yaks was calculated by dividing the number of yaks slaughtered in the year-end by the number of yaks from the previous year. Data on meat production [25], number of yaks slaughtered [26] and number of year-end yaks [27] in 44 counties of Qinghai Province in 2018 were provided by the Agricultural and Rural Department of Qinghai Province [24, 26, 27] and collected through the National Tibetan Plateau Data Center (http://data.tpdc.ac.cn). Annual average county-level oxygen concentrations (%) in Qinghai Province were obtained from Hu [28]. A univariate linear regression equation was constructed to analyze the relationship between yak meat production, yak slaughter rate and annual oxygen concentration. The average annual oxygen concentration was used as an independent variable, while meat production per yak and yak slaughter rate were used as dependent variables.

### Animal feeding and sampling

Ten cattle (*Bos taurus*, initial weight = 245.5 ± 50 kg) and ten yaks (*Bos grunniens*, initial weight = 211.6 ± 40 kg) were procured from Datong County (36.55°N, 101.42°E, 2400 m above sea level: m ASL), Xining City, Qinghai Province. The experiment was divided into two periods of 28 days each. The first part of the experiment was conducted in May 2021 at Qinghai Academy of Animal Science and Veterinary Science (36.65°N, 101.77°E, 2200 m ASL). For the second part of the experiment, the same animals were transported to Xueduo Yak Breeding Base, located in Henan County in the Huangnan Autonomous Prefecture of Qinghai Province (34.73°N, 101.62°E, 3800 m ASL) in June 2021. The animals were kept in individual cages (1.5 × 3 m) in an open-sided roofed structure throughout the study. Oat hay was provided *ad libitum* twice daily at 08:00 and 17:00, and drinking water was available round the clock. Daily feed intake was calculated by subtracting the feed refused from the feed offered. The first 21 days at each location were considered as the adaptation period, followed by 7 days for data collection. At the end of each period, the animals were weighed and blood samples were collected from the jugular vein using vacuum tubes with anticoagulant, before morning feeding. The blood was centrifuged at 3000 x *g* for 20 min to obtain serum. Part of the serum sample was stored at -20℃ for analysis of blood biochemical indices, while the remaining part was stored at -80℃ for determination of blood metabolites. Rumen fluid was collected using a flexible oral stomach tube with a metal strainer, which was washed with clean warm water after each collection. The first 50 mL of fluid was discarded to avoid saliva contamination. The rumen fluid was filtered through four layers of gauze, and the pH was measured immediately using a pH meter (Ecoscan pH 5, Singapore). The rumen fluid was stored immediately in liquid nitrogen for determination of fermentation parameters and microbial extraction.

### Evaluation of apparent indicators

Fecal samples were collected at 08:00 and 16:00 for four consecutive days at the end of each experimental period. After mixing, 10% of the feces was divided into two parts: one part was treated with 10 mL of 10% sulfuric acid and stored at -20℃ for determination of CP; while the other part was dried at 65℃ for 48 h until a constant weight was achieved and saved for proximate analysis. Feed samples were collected on two consecutive days during each week, mixed and stored at -20 °C for subsequent determination of their nutrient composition. Dry matter and total N) were analyzed according to the procedure 976.05 of AOAC [29]. The NDF and ADF were determined by the method of Van Soest et al. [30] using an ANKOM 200i fiber analyzer (ANKOM Technologies, Inc., Fairport, NY, USA). The hemicellulose was calculated by the difference between NDF and ADF [31]. The serum concentrations of aspartate aminotransferase (AST), alanine aminotransferase (ALT), total protein (TP), albumin (ALB), globulin (GLOB), blood glucose (Glu), total cholesterol (TCHO), triglyceride (TRIG), alkaline phosphatase (ALP), total bilirubin (TBIL), creatine kinase (CK), lactate dehydrogenase (LDH), UREA, uric acid (UA), Ca, and P were determined using an automatic blood biochemical analyzer (SRL, Inc., Tokyo, Japan). Immunoglobulin A (IgA) and immunoglobulin M (IgM) were measured using commercial ELISA kits (Shanghai Enzyme-linked Biotechnology Co., Ltd, Shanghai, China).

### Measurements of rumen fermentation parameters

Short chain fatty acids (SCFAs: acetate, propionate, isobutyrate, butyrate, isovalerate, valerate) were measured by gas chromatography using an Agilent 7890B system (Agilent, Santa Clara, CA, United States). The column temperature injection temperature and the TCD (Thermal Conductivity Detector) temperature were maintained at 40, 220 and 230 °C, respectively, following the method described by Li et al. [32]. Air, nitrogen carrier gas, and hydrogen were maintained at a pressure of 0.05 MPa. Crotonic acid was used as the internal standard for calculating the concentrations of the SCFAs, and standard curves were established. The concentration of NH_3_-N in the rumen fluid was measured using the colorimetric method following the methods of Shen et al. [33]. Rumen microbial crude protein (MCP) was measured using Bradford Protein Assay Kit (Beijing Solarbio Science and Technology Company, Beijing, China) using Coomassie brilliant blue as the dye [34, 35].

### DNA extraction, 16 S rRNA gene amplicon sequencing, and high-throughput sequencing

#### DNA extraction and amplicon sequencing

The total DNA from rumen fluid was extracted using the Cetyltrimethylammonium bromide (CTAB) method described by Dai et al. [36]. The DNA concentration was measured using a Nanodrop ND-1000 spectrophotometer (Thermo Fisher Scientific, Waltham, MA, USA). The total bacterial 16 S rRNA gene was amplified using the primers 515-F (5”-GTGCCAGCMGCCGCGGTAA-3”) and 806-R (5”-GGACTACCVGGGTATCTAAT-3”) using PCR thermal cycler (Eppendorf AG 22331 Hamburg, Germany). After purification using Agencourt AMPure XP magnetic beads (Beckman Coulter, Milan, Italy), library quality was assessed on the Illumina Hiseq platform at BGI Life Tech Co., Ltd. (Beijing, China) before sequencing. Ambiguous and low-quality sequences were removed using Cutadapt v2.6 software [37], and paired reads were assembled using the sequence splicing software Flash v1.2.11 [38]. This was used to obtain tags in the hypervariable region based on the overlap relationship. Operational taxonomic units (OTUs) were clustered into different characteristic sequences (Features) using the Vsearch plug-in in QIIME2 [39] with a 99% similarity cutoff standard. Rarefaction curves were obtained using QIIME2 diversity (Supplementary Fig. S1A). The relative abundances of rumen bacteria at the phylum and genus levels were determined by annotating with the Silva 16 S rRNA gene database SILVA_138. The α and β diversities were analyzed using QIIME2 software, and principal coordinate analysis (PCoA) plots were generated using ggplot2.

#### DNA extraction and amplicon sequencing

A normalization method was employed to minimize biases arising from differences in scales of the original data and to enable subsequent comparisons between yaks and cattle for identifying differential microorganisms. Specifically, given the original data points $\:{x}_{1},{x}_{2},\cdots\:,{x}_{n},$ the 𝑖 -th normalized data point was defined as

$$\:\widehat{{x}_{1}}=\frac{{x}_{i}}{{\sum\:}_{j=1}^{n}{x}_{j}}.$$

Here, the denominator $\:{\sum\:}_{j=1}^{n}{x}_{j}$ represents the sum of all data values. This normalization method transformed the data into [0, 1] range, preserving the relative proportions among data points while eliminating differences in absolute values.

Differential microorganisms were subjected to paired Wilcoxon test after normalization to obtain p-values. The results were considered significantly different at *P* < 0.05. Based on this significance level, the Omitted group was determined by *P* > 0.05 and abs (Log10FC) < 0.301.

#### Metagenomic assembly and functional annotation

After processing the samples as described above, the purified metagenomic samples were sent to Illumina Hiseq platform at BGI Life Tech Co., Ltd. (Beijing, China) for high-throughput sequencing. For quality control, the raw sequencing data were processed using Trimmomatic (v.0.39) [40]. Low-quality bases and adapter sequences were removed from the raw reads, and the quality was evaluated using FastQC (v.0.12.1) (https://www.bioinformatics.babraham.ac.uk/projects/fastqc/). The filtered reads were matched with the reference genome using BWA (v.0.7.17, r1188) [41] at default settings. Reads that matched with the host genome were removed using Samtools (v.1.21) [42] to ensure that only non-host sequences were retained for downstream analysis. The filtered, host-free sequences were assembled *de novo* using MEGAHIT (v.1.2.9) [43] with default settings.

For functional annotation, eggNOG-mapper (v2.1.12) [44] was employed, using the eggNOG database version 5.0.2, along with Diamond (v2.1.9) [45] and MMseqs2 (v15.6f452) [46] to map functional categories. The annotation was performed with the ‘--itype metagenome’ parameter, enabling the identification of functional genes and pathways within the metagenomic dataset.

#### Species-pathway integration

Filter KEGG pathway information from Eggnog results, using Python, was collected and the statistics was summarized to obtain pathway information for each sample and the corresponding read names. Normalization was performed using the same method as employed during the amplification step. After normalization, a paired Wilcoxon test was performed to obtain differential pathway information (*P* < 0.05). Based on these reads, corresponding assembled sequences were extracted to annotate the species composition of these differentially expressed pathway sequences. Kraken2 databases were constructed using a previously established MAGs library. The protozoa were used to generate NCBI taxon IDs “40635”, “47888”, “5986”, “47895”, “40637”, “358016” and the database of Li et al. [47]. Viruses employed the database of Yan and Yu [48]. Anaerobic fungi genomic data was incorporated from the studies of Pratt et al. [49], Haitjema et al. [50], Brown et al. [51], Mondo et al. [52], Wilken et al. [53], Youssef et al. [54] and Li et al. [55], while for bacteria and archaea we used the database of Xie et al. [56]. Kraken2 (v2.1.3) was used at the ‘--fast-build’ option to enable rapid database construction, specifically tailored to the rumen microbiome as described by Wood et al. [57]. Species-level taxonomic annotations were performed on the assembled metagenomic reads data containing differential pathways. The obtained species information was merged, and the annotated species data based on group mean values, were visualized through pie charts and microbial interaction networks. The process flowchart is shown in Supplementary Fig. S2. The WGS and amplicon information is shown in Supplementary Table S1, 2.

### Real-time quantitative PCR

Total RNA was extracted using the RN43-EASYspin Plus Kit (Aidlab Biotech Co., Ltd. China) according to the manufacturer’s protocol, and the quality of the RNA was assessed using a Nanodrop ND-1000 spectrophotometer (Thermo Scientific, Waltham, MA, USA). The RNA was, then, reverse-transcribed into cDNA using the TRUEscript RT MasterMix Kit (Aidlab Biotech Co., Ltd. Beijing, China). Real-time quantitative PCR (qPCR) was used to analyze the change in total bacterial copy number. The PCR premix contained 10 *µ*L of SYBR^®^ Green Pro Taq HS Premix (Rox Plus) (Accurate Biotechnology Co., Ltd, Hunan, China), 0.4 *µ*L of forward primer (CCTACGGGAGGCAGCAG), 0.4 *µ*L of reverse primer (ATTACCGCGGCTGCTGG), 2.0 *µ*L of DNA template, and 7.2 *µ*L of nuclease-free water (Accurate Biotechnology Co., Ltd, Hunan, China) in a 20 *µ*L reaction system. The thermocycling conditions were maintained as follows: Temperature of 95 °C was maintained for 30 s for denaturation and activation of Taq polymerase, followed by 40 thermal cycles at 95 °C for 5 s and then 60 °C for 30 s. After the amplification process, melting curve analysis was conducted at 95 °C for 15 s and 60 °C for 60 s for the dissociation stage. The fluorescence was detected by the QuantStudio 5 Real-time PCR Instrument (Thermo Fisher Scientific, Waltham, MA, USA).

### Blood metabolomics analysis

The procedures for metabolomics analyses were followed as described by Cox et al. [58]. Briefly, each sample of 100 *µ*L of serum was mixed with 300 *µ*l methanol (-20 °C) in 1.5 mL centrifuge tubes and left at -20 °C for one hour. After centrifuging at 10,000 x g at 4℃ for 15 min, 5 *µ*L of internal standard (1 *µ*g/mL, DL-o-Chlorophenylalanine) was added to 200 *µ*L of supernatant. It was then transferred to a vial for liquid chromatography-mass spectrometry (LC-MS) analysis using the LC-MS platform (Q Exactive Thermo Fisher Scientific, Walthem, MA, USA). The chromatographic separation was carried out using an ACQUITY UPLC HSS T3 column (100 × 2.1 mm 1.8 *µ*m) at 40 °C with a flow rate of 0.3 mL/min. The injection volume was 6 µL, and the automatic injector temperature was 4 °C.

Data on retention time, compound molecular weight, observations and peak intensity were collected using feature extraction, preprocessed with compound discoverer software (Thermo) and normalized. To ensure data quality, peaks from less than 50% of QC samples and 80% of biological samples were discarded. The OPLS-DA was employed to visualize the overall differences and for identifying differential metabolites. The Kruskal‑Wallis nonparametric test was applied to compare metabolite abundances among groups. Fifferential metabolites were selected according to the importance of variable in projection (VIP), false discovery rate (FDR), *P*-value and Log2 fold change (Log2FC) (VIP > 1, FDR < 0.05, *P*-values < 0.05, abs (Log2FC) > 0.263).

### Statistical and data visualization analysis

Statistical analyses were performed in R (version 4.3.3) [59]. The Kruskal-Wallis nonparametric test was used to compare differences in rumen microbial communities. For further statistical analysis and visualizations, the following R packages were used: ggtree (version 3.16.0) [60], ggtreeExtra (version 1.18.0) [61], ggplot2 (version 3.5.2) [62], tidyverse (version 2.0.0) [63], treeio (version 1.32.0) [64], dplyr (version 1.1.4) [65], reshape2 (version1.4.4) [66], ggnewscale (version 0.5.1) [67], viridis (version 0.6.5) [68], ggpubr (version 0.6.0) [69], rjson (version 0.2.23) [70], and ggrepel (version 0.9.6) [71, 72]. Data were presented as mean ± standard error of the mean (SEM), and significance was set at *P* < 0.05.

In addition, Python (version 3.13.0) was employed for specific tasks, including the retrieval of KO information using the requests library. ETE4 (version 4.3.0) [73] was used for constructing phylogenetic trees, while SciPy (version 1.15.3) [74] and Pandas (version 2.2.3) [75] were used for additional data manipulation and analysis.

## Results

### Environmental parameters on Qinghai-Tibetan Plateau

Variation in altitude resulted in simultaneous changes in multiple environmental factors. Thus, the sunrise time (SRT) advanced (*P* < 0.05) and the sunset time (SST) was delayed (*P* < 0.05), resulting in a marked increase in daylight duration (DLD) at higher altitudes. However, the ultraviolet (UV) index showed no difference (*P* > 0.05, Supplementary Fig. S3A). The maximum temperature (MaxT) and daily temperature range (DRT) increased (*P* < 0.05) with increasing altitudes; however, the minimum temperature (MinT) was not affected (*P* > 0.05, Supplementary Fig. S3D). The oxygen levels (Supplementary Fig. S3A), air pollution index (API), PM10, PM2.5, NO_2_, SO_2_, and CO (Supplementary Fig. S3B), water dissolved oxygen (WDO), water temperature (WT), and water pH (WpH) (Supplementary Fig. S3E) decreased (*P* < 0.05), and the humidity (AH) and ozone (O_3_) increased (*P* < 0.05) with increasing altitude. The barn temperature (BT) decreased (*P* < 0.05), while the humidity (BH) increased (*P* < 0.05, Supplementary Fig. S3F) with increasing altitudes.

### Hypoxia reduces nutrient digestibility

Food intake (FI) of both cattle and yaks increased (*P* < 0.05, Supplementary Fig. S4A, B), and metabolic body weight (MBW) and respiratory rate (RR) of yaks decreased (*P* < 0.05, Supplementary Fig. S4A, B) with increasing altitude. The chemical and nutritional composition e of the experimental diets are presented in Table 1 and the digestibility parameters are presented in Table 2. The digestibilities of DM, OM, CP decreased with increasing altitudes both in yaks and cattle (*P* < 0.05). The apparent dry matter fermentability (ADFD) decreased in yaks (*P* < 0.05, Supplementary Fig. S4C), (Supplementary Fig. S4C) but not in cattle (*P* > 0.05). These shifts in nutrient digestibility serve as the initial physiological response to the high-altitude environment, closely related to subsequent alterations in rumen fermentation dynamics.

**Table 1:** Nutrient composition of experiment feed (%, DM basis)

Nutrient composition	Content
DM	92.2
CP%	5.1
NDF%	65.7
ADF%	37.8
Ash	6.4

**Table 2:** Nutrient digetibility in yaks and cattle maintained at low and high altitudes

Items	Yaks		*P* value	Cattle		*P* value	(H-L)/L*100%		*P* value
	Low	High		Low	High		Yaks	Cattle	
Feed intake (g/kgW^0.75^)	53.0 ± 2.1	59.5 ± 3.1	0.041	89.9 ± 3.6	103.8 ± 3.6	0.022	13.1 ± 5.5	17.5 ± 7.4	0.638
Water intake (g/kgW^0.75^)	153.6 ± 10.8	174.2 ± 10.8	0.134	315.5 ± 15.7	294.9 ± 11.3	0.333	17.1 ± 9.7	-4.3 ± 6.2	0.079
DMD (%)	66.1 ± 1.2	57.1 ± 0.4	< 0.001	57.4 ± 1.4	51.4 ± 0.9	0.002	-13.3 ± 2.0	-10.1 ± 2.0	0.274
OMD (%)	70.7 ± 1.2	62.8 ± 0.4	< 0.001	62.1 ± 1.4	56.9 ± 0.9	0.007	-10.9 ± 1.9	-8.1 ± 1.8	0.292
NDFD (%)	67.2 ± 1.4	64.0 ± 1.0	0.078	56.6 ± 1.5	54.1 ± 1.5	0.255	-5.1 ± 3.5	-2.0 ± 2.8	0.503
ADFD (%)	66.5 ± 1.2	61.5 ± 0.9	0.003	55.4 ± 1.6	51.5 ± 1.4	0.083	-7.1 ± 2.9	-4.0 ± 2.8	0.448
CPD (%)	51.0 ± 2.0	20.2 ± 1.9	< 0.001	46.8 ± 1.5	25.7 ± 2.9	< 0.001	-60.0 ± 3.8	-45.5 ± 5.7	0.049
NDSD (%)	68.7 ± 0.8	51.8 ± 1.4	< 0.001	66.6 ± 1.1	54.8 ± 2.5	< 0.001	-24.5 ± 2.0	-17.4 ± 4.3	0.157

### Hypoxia increases ammonia nitrogen levels in yaks

**Fig. 1:**
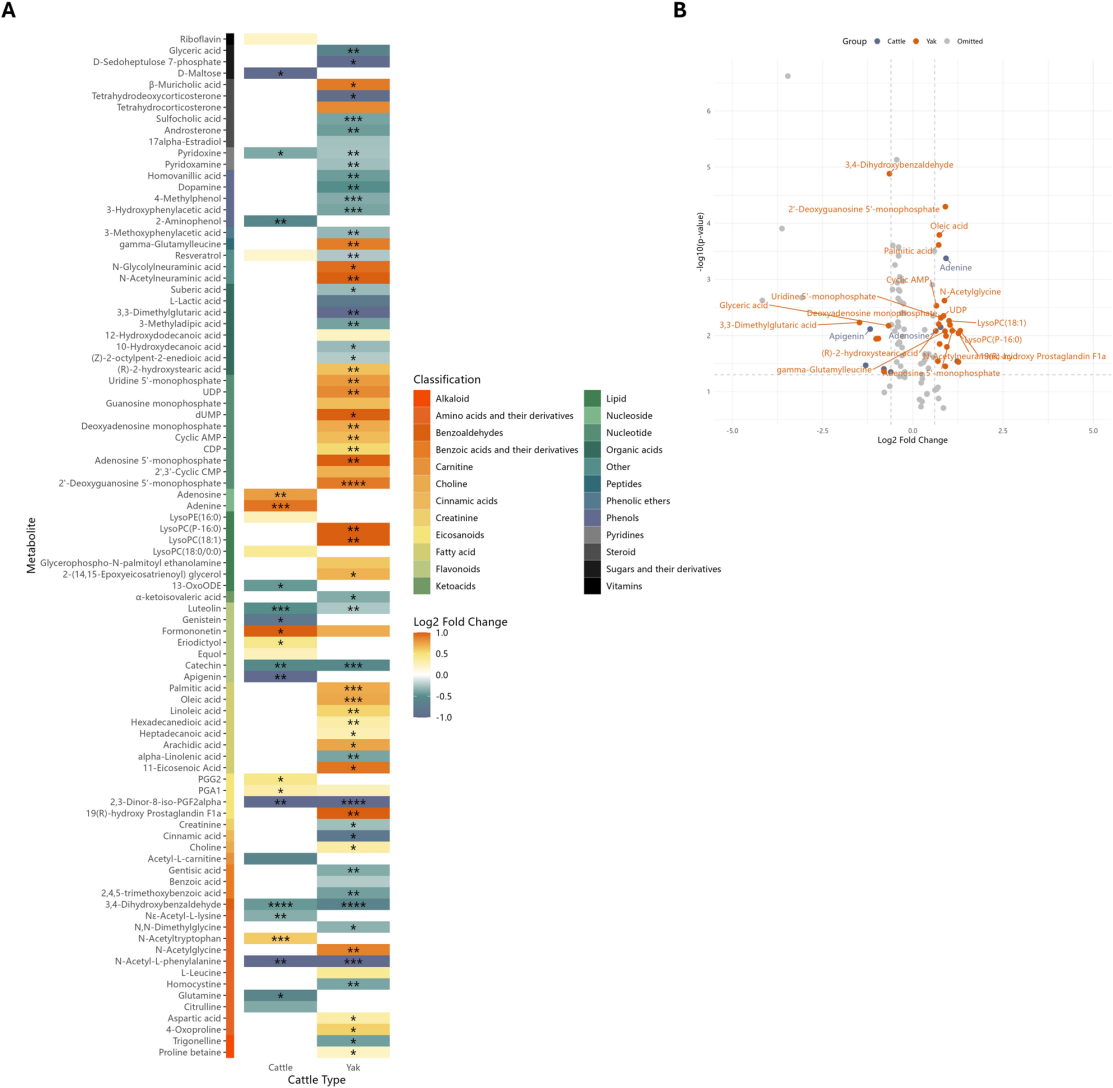
Rumen metabolomics results: (**A**) Comparison of differential metabolites between yaks and cattle; (**B**) Volcano plot comparison of differential metabolites between yaks and cattle, where the ‘Omitted’ group represents metabolites present in both groups or *P* > 0.05 or Log2FC < 0.606. Significant differences between low- and high-altitude regions are denoted as follows: *p < 0.05, **p < 0.01, ***p < 0.001, ****p < 0.0001

Driven by changes in digestibility and microbial community structure, the metabolic functions within the rumen were significantly altered. With increasing altitude, microbial crude protein (MCP) synthesis decreased in both cattle and yaks (*P* < 0.05; Supplementary Fig. S5A). As compared with cattle, the concentration of ammonia nitrogen (NH3-N) was higher in yaks at higher altitudes (*P* < 0.05; Supplementary Fig. S5A). In cattle, the concentrations of total volatile fatty acids (TVFA; Supplementary Fig. S5B), isobutyric acid (IBA), propionic acid (PA), and acetic acid (AA) increased with increasing altitude (*P* < 0.05). IBA levels also increased in yaks (*P* < 0.05; Supplementary Fig. S5C). Furthermore, no differences were observed in the level of butyric acid (BA) between cattle and yaks (*P* > 0.05; Supplementary Fig. S5C). Additionally, fatty acid and nucleotide metabolism was enhanced in yaks (Fig. 1A), which exhibited a greater number of metabolite changes with increasing altitude (Fig. 1B; Supplementary Tables S3 and S4).

### Hypoxia reduces microbial protein synthesis and alters rumen metabolite profiles in yaks

**Fig. 2:**
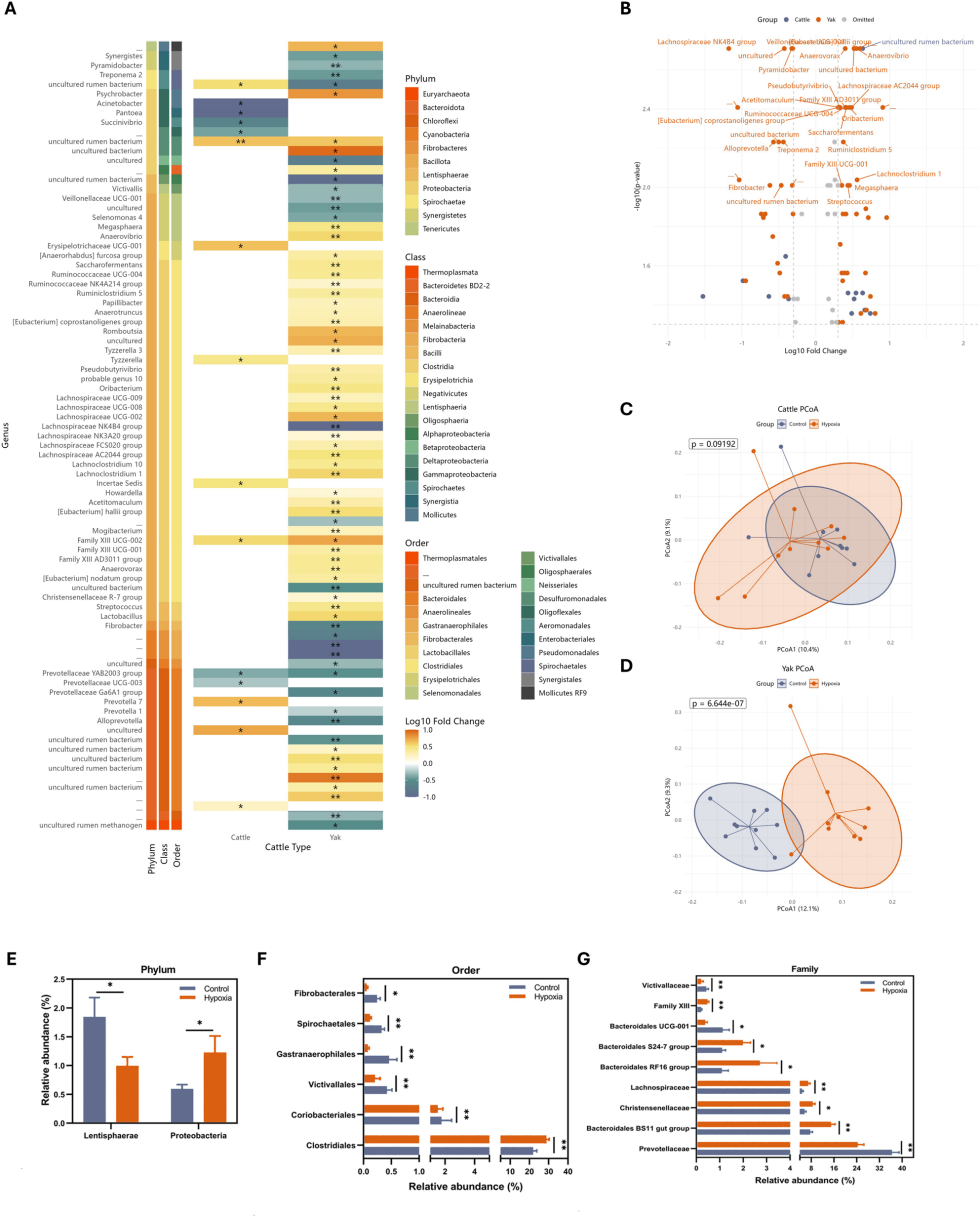
Rumen microbial difference results: (**A**) Comparison of 16S differential microorganisms between yaks and cattle; (**B**) Volcano plot comparison of 16S differential microorganisms between yaks and cattle, where the ‘Omitted’ group represents metabolites present in both groups or *P* > 0.05 or Log2FC < 0.606; (**C**) Principal coordinate analysis (PCoA) of rumen bacterial community structure in cattle based on unweighted UniFrac dissimilarity; (**D**) Principal coordinate analysis (PCoA) of rumen bacterial community structure in yak based on unweighted UniFrac dissimilarity; (**E**) Differential rumen bacteria at the phylum level in yak; (**F**) Differential rumen bacteria at the order level in yak; (**G**) Differential rumen bacteria at the family level in yak. Differential abundance is indicated by asterisks representing statistical significance: *p < 0.05, **p < 0.01

To elucidate the biological drivers behind the observed changes in metabolic functions, we analyzed the rumen microbiome. High-throughput 16 S rRNA gene amplicon sequencing analysis of the rumen microbiome revealed that the bacterial community structure in yaks exhibited significant changes with varying oxygen levels. In contrast, no significant structural changes were observed in cattle (Fig. 2A, B), with varying oxygen levels (*P* < 0.05, Fig. 2C, D).

At the phylum level, the hypoxic yak group exhibited a lower relative abundance of Lentisphaerae (*P* < 0.05) and a higher relative abundance of *Proteobacteria* (*P* < 0.05) than control (Fig. 2E). At the order level, differences were observed in the relative abundance of Fibrobacterales, Spirochaetales, Gastranaerophilales, Victivallales, Clostridiales, and Coriobacteriales in yaks (*P* < 0.05, Fig. 2F).

**Fig. 3:**
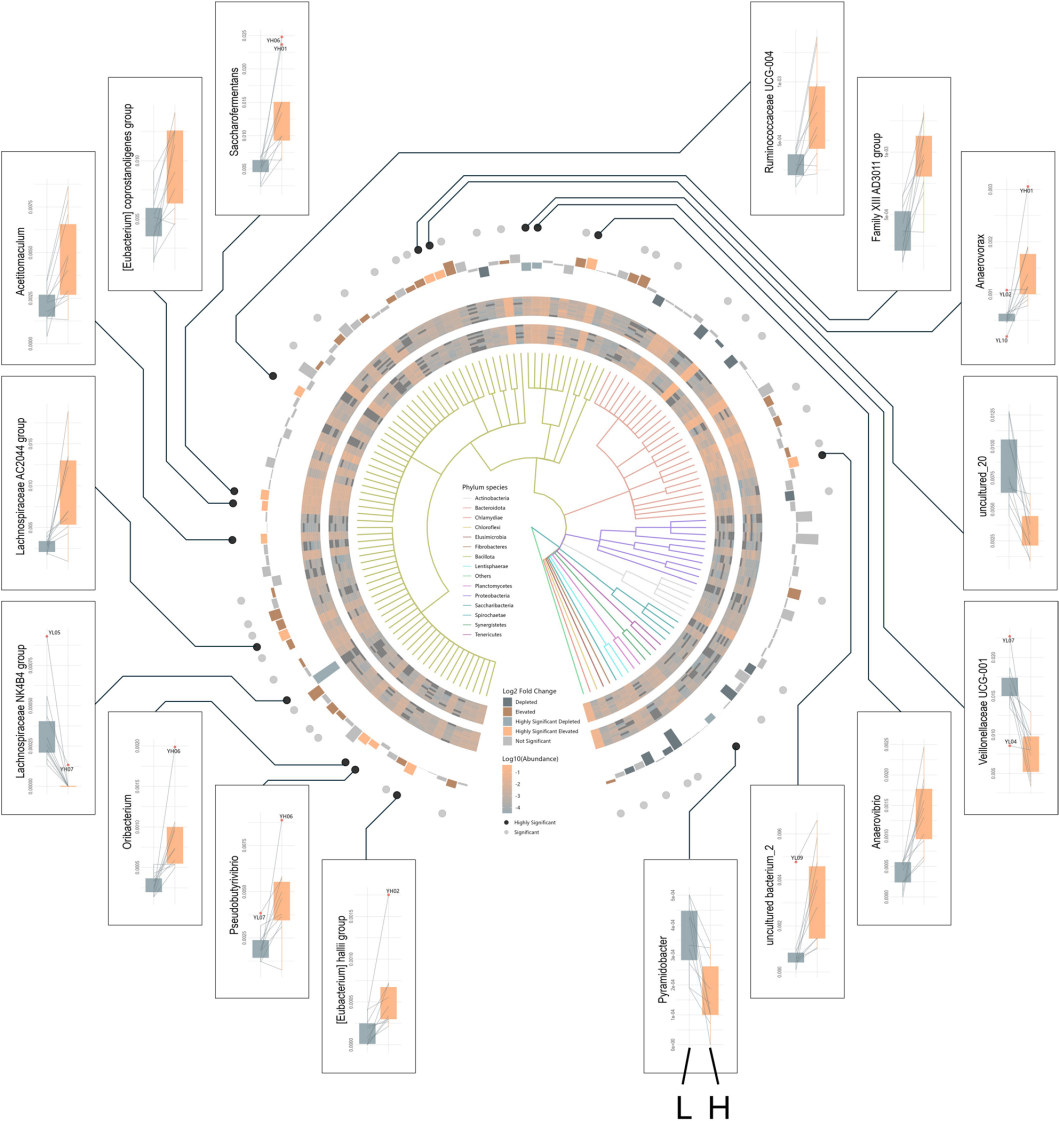
Rumen microbiota composition in cattle. The phylogenetic tree illustrates the abundance and diversity of species, predominantly belonging to Actinobacteria, Bacteroidetes, Firmicutes, Fusobacteria, Lentisphaerae, Planctomycetes, and Spirochaetae; Outer ring: Species with significantly increased or decreased abundance (*p* < 0.005) are marked with black dots, accompanied by corresponding boxplots. Boxplots show abundance values on the x-axis, with light blue indicating abundance at low altitude and light red at high altitude; Second outer ring: Log2 fold change (log2FC) analysis of species abundance. Deep red and deep blue indicate significantly increased or decreased abundance (*p* < 0.005), while light red and light blue indicate significant changes at *p* < 0.05༛Inner two rings: Abundance profiles of species in samples from low- and high-altitude regions (log10-transformed abundance). The inner circle represents low-altitude samples, and the outer circle represents high-altitude samples

At the family level, hypoxia induced changes in the relative abundance of Prevotellaceae, Bacteroidales BS11 gut group, Christensenellaceae, Lachnospiraceae, Bacteroidales RF16 group, Bacteroidales S24-7 group, Bacteroidales UCG-001, Family XIII, and Victivallaceae in yaks (*P* < 0.05, Fig. 2G). Genus level analysis identified 53 differential taxa (*P* < 0.05), including several unclassified groups as ‘others,’ with 16 taxa showing highly significant differences (*P* < 0.005, Fig. 3) at varying oxygen levels.

In cattle, only six genera showed differences in genus (*P* < 0.05), including one unclassified rumen bacterium (*P* < 0.005, Fig. 2A) at varying oxygen level (Supplementary Table S5 and S6).

Rumen metabolome analysis combined with volcano plot (Fig. 1B) filtering identified hypoxia‑responsive metabolites in yaks but not in cattle. In yaks, long‑chain fatty acids including LysoPC(18:1) and LysoPC(16:0) were increased under hypoxic conditions compared with controls (*P* < 0.05). Several nucleotides, such as cyclic AMP, CDP, and UDP, also showed increased abundance in hypoxic yaks (*P* < 0.05). None of these metabolites were significantly altered in cattle under the same oxygen treatment (*P* > 0.05).

### Hypoxia reduced immunity and increased the inflammatory factors

The effects of environmental stress, microbial restructuring, and altered rumen metabolism affected host physiological and immune indicators. Total Bilirubin (TBIL) decreased (*P* < 0.05, Supplementary Fig. S6A), and glucose (GLU) level increased (*P* < 0.05, Supplementary Fig. S6A) both in in cattle and yaks under hypoxic condition. Alanine Aminotransferase (ALT) increased in yaks (*P* < 0.05, Supplementary Fig. S6A) but not in cattle.

**Fig. 4:**
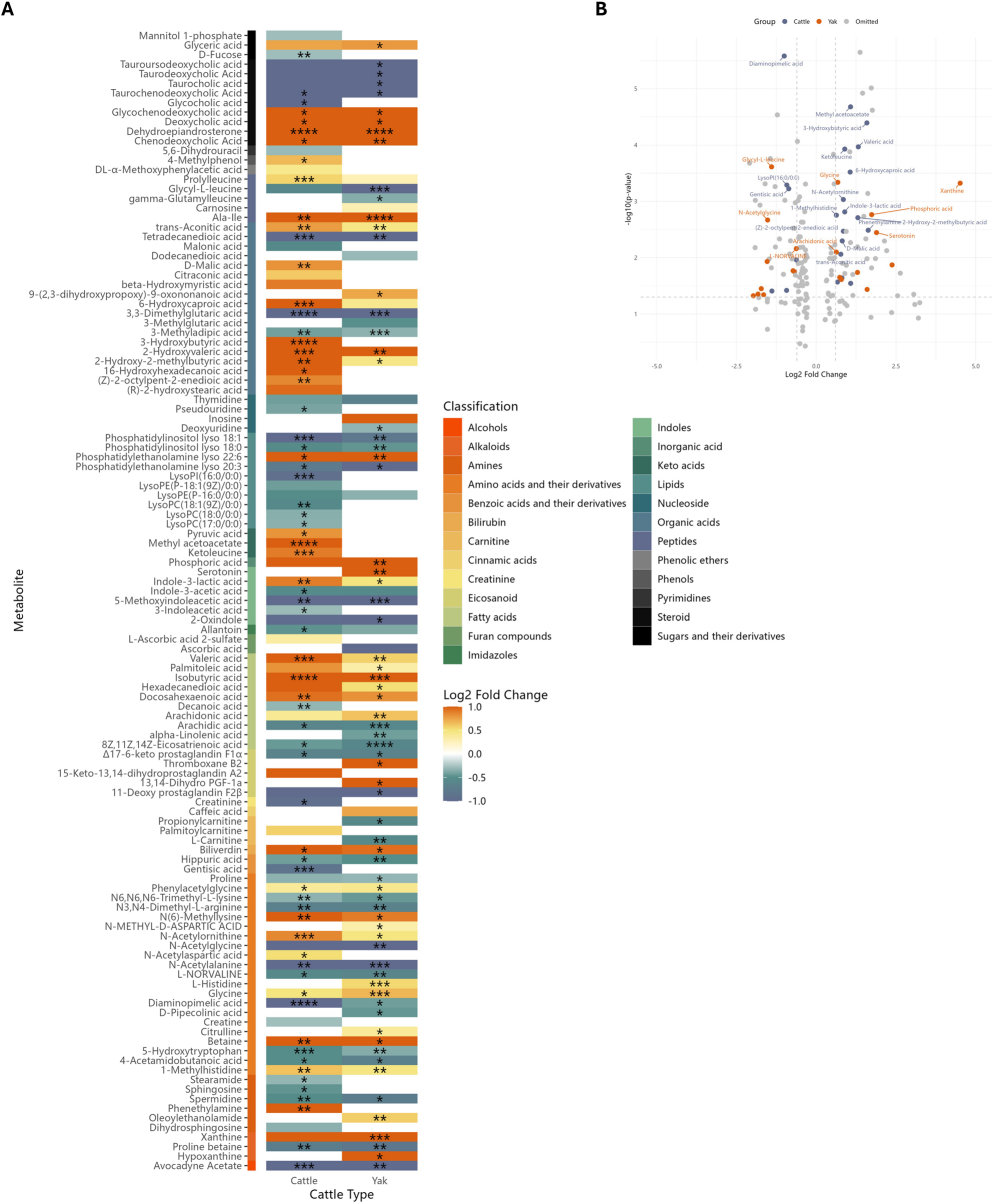
Blood metabolomics results: (**A**) Comparison of differential metabolites between yaks and cattle; (**B**) Volcano plot comparison of differential metabolites between yaks and cattle, where the ‘Omitted’ group represents metabolites present in both groups or *P* > 0.05 or Log2FC < 0.606. Significant differences between low- and high-altitude regions are denoted as follows: *p < 0.05, **p < 0.01, ***p < 0.001, ****p < 0.0001

Blood immunity, interleukin (IL), malondialdehyde (MDA), oligosaccharide (OGA), and immunoglobulin M (IGM) increased both in cattle and yaks (*P* < 0.05, Supplementary Fig. S6B). No differences were observed in tumor necrosis factor (TNF, *P* > 0.05, Supplementary Fig. S6B) both in yaks and cattle. Yaks showed fewer changes in the blood metabolome (Fig. 4A, B) than in cattle (Supplementary Table S7, S8 and S9) under hypoxic conditions.

### Hypoxia alters the functions of the yak’s body and rumen microorganisms

**Fig. 5:**
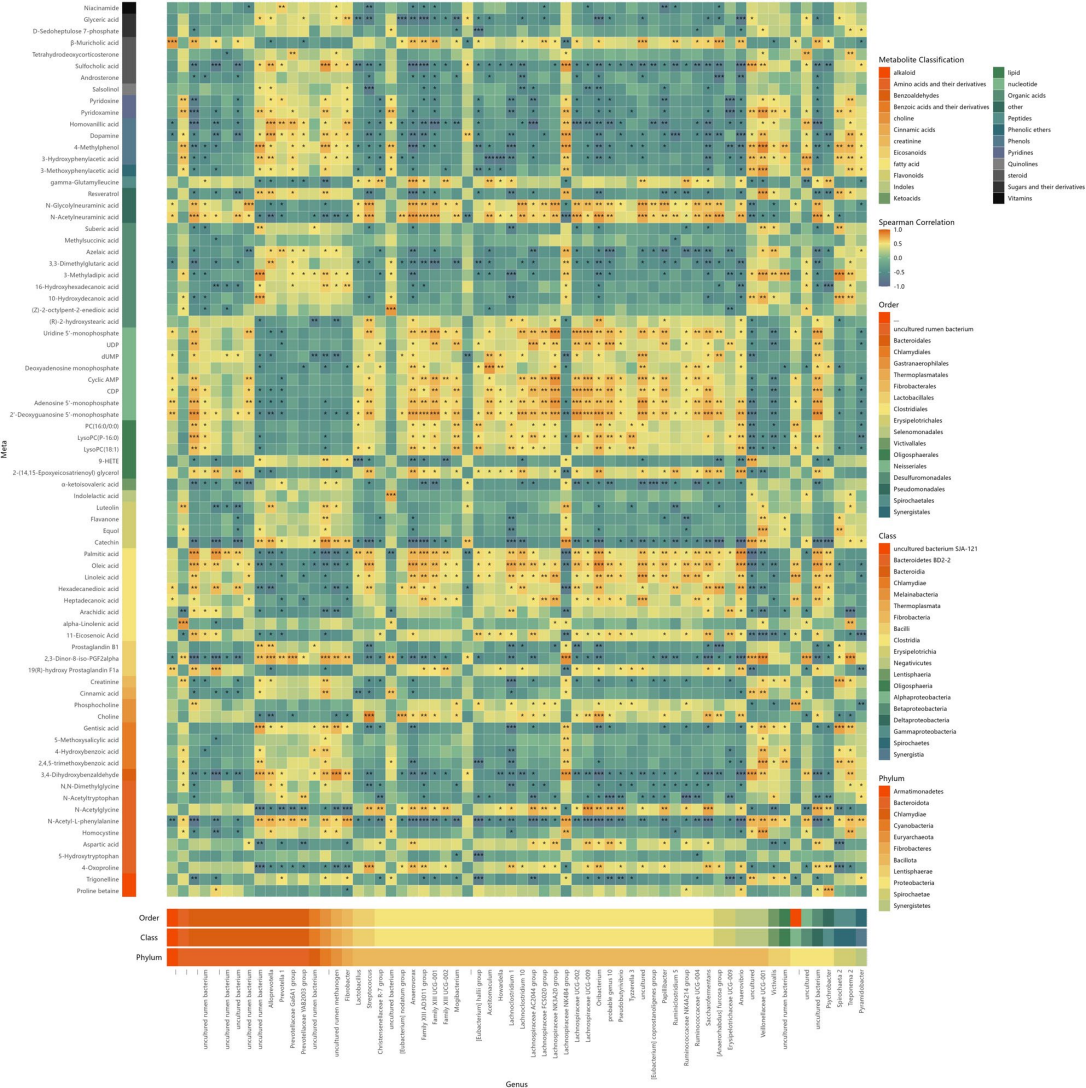
Heatmap of correlation between differential microorganisms and differential metabolites in yak rumen. The Y-axis represents differential metabolites, with colored blocks distinguishing different metabolite categories, and the X-axis represents differential microorganisms at the genus level, with colored blocks distinguishing different level classifications. The heatmap colors indicate the Spearman correlation of differential metabolites. Significant differences between low- and high-altitude regions are denoted as follows: *p < 0.05, **p < 0.01, ***p < 0.001

**Fig. 6:**
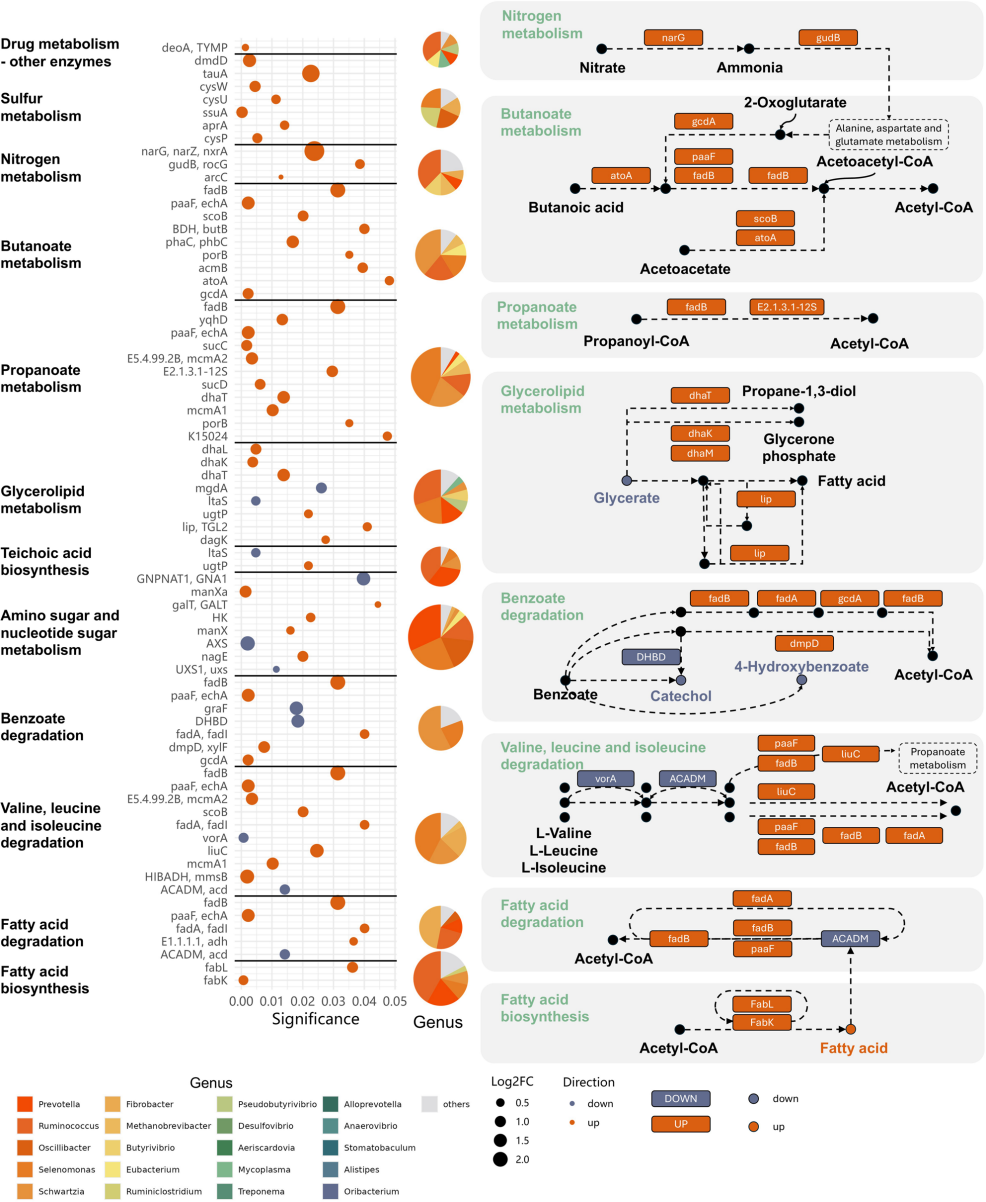
Summary of changes in rumen microbial functional genes in KO genes and KEGG pathway modules. The left bubble chart shows the KOs and MAPs corresponding to the differential KO numbers in functional annotations of microbes from both regions, the pie chart displays the abundance information of species annotations for reads from differential KOs in the corresponding left MAP, and the right side presents detailed pathway results of the main MAP functions along with their increase or decrease status

**Fig. 7:**
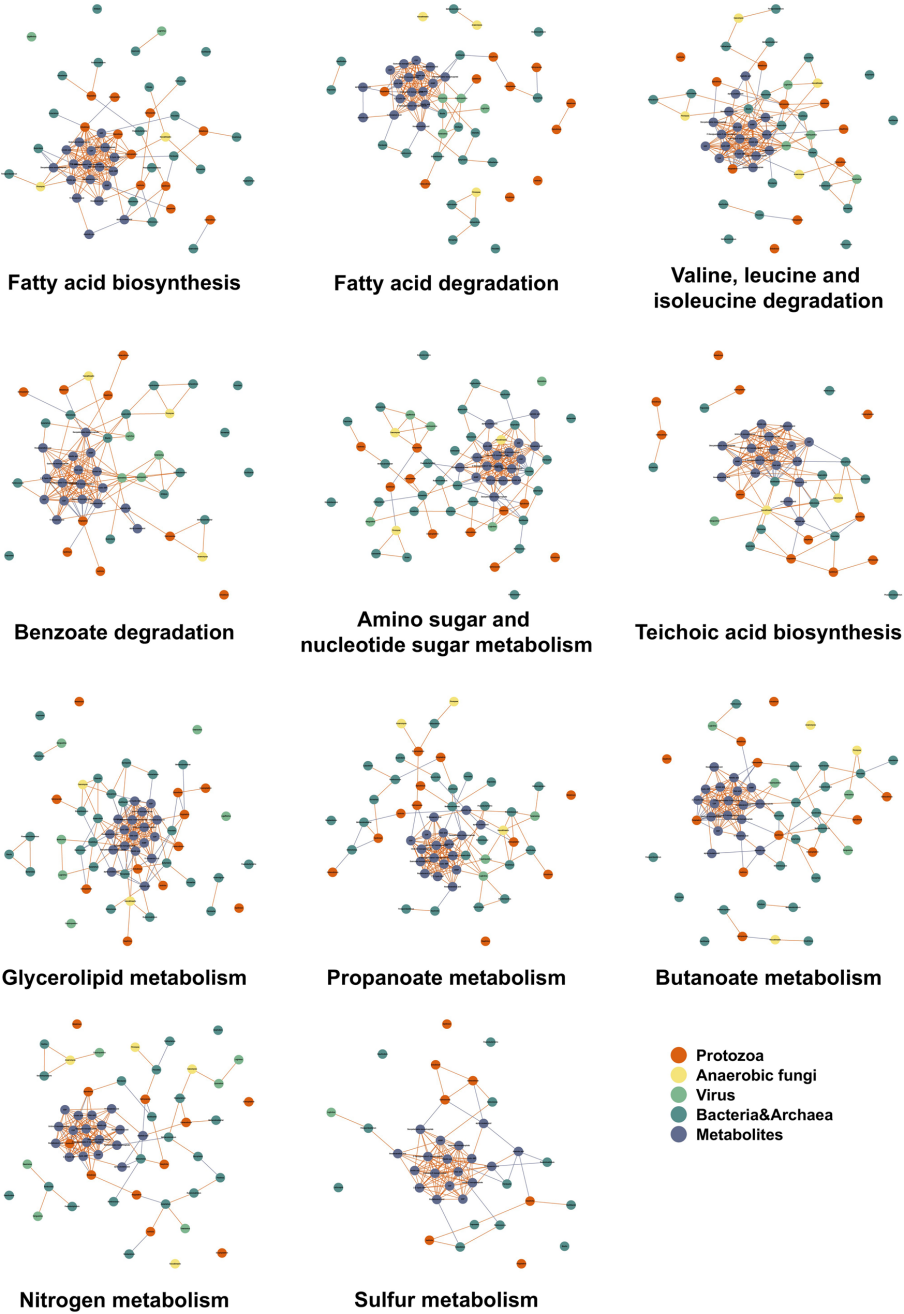
Microbial associated network of the pathway map of interest. Red edges represent positive correlations, while blue edges represent negative correlations (abs(correlation) > 0.5)

In correlation analysis of differential metabolites and differential microorganisms in the rumen of yaks, it was observed that Bacillota and Bacteroidota phyla were positively correlated with fatty acids, nucleotides, and their derivatives (Fig. 5, Supplementary Table S3). Microbial functions in the rumen were enriched during the biosynthesis pathways of unsaturated fatty acids, primary bile acid biosynthesis, and serine and threonine metabolism (Supplementary Fig. S1B). Functional annotation of rumen microorganisms using eggnog showed that the involved in pathways were mainly enriched in fatty acid biosynthesis; valine, leucine and isoleucine degradation; amino sugar and nucleotide sugar metabolism; nitrogen metabolism; propanoate metabolism; butanoate metabolism and related biological processes. In the species annotation of functional reads, *Ruminococcus*, *Oscillibacter*, *Selenomonas*, *Schwartzia* and *Fibrobacter* were the main contributors to microbial functions (Fig. 6). Microbial interaction networks (Fig. 7) analyses across various pathways revealed that distinct pathways are associated with different sets of microbial taxa (Supplementary Table S10 and S11).

## Discussion

Although yaks possess distinct hypoxia-tolerant traits [19]; However, in this study, decreasing air oxygen levels was associated with significant reductions in slaughter rate and meat yield of local yaks (Supplementary Fig. S7A, B). These findings suggest that while traditional genetic adaptation theories form a critical foundation for understanding high-altitude survival in yaks, they may not fully account for all the complex mechanisms at play, highlighting the complementary role of microbial-mediated physiological adjustments. Based on this comparative study of cattle and yaks, we identified distinct environment-associated adaptive features in the yak rumen, including interconnected processes of restructuring of the microbial community, specialization of metabolic pathways, and host-microbe co-regulation. A distinct adaptive metabolic pattern centered on long-chain fatty acid metabolism, suggests that biosynthesis pathways, potentially mediated by the genera *Ruminococcus*, *Oscillibacter*, *Selenomonas*, *Schwartzia*, and *Fibrobacter* may form the functional core of this process (Fig. 6). This metabolic shift was closely associated with markers of hepatic stress, highlighting the physiological cost of high-altitude adaptation in yaks. Notably, this association is based on correlative evidence and requires further functional validation to confirm causal relationships between the observed metabolic shift and hepatic stress markers.

The hypoxic, high-altitude environment is associated with distinct shifts in the rumen microbiota of yaks. First, under both high- and low-altitude conditions, the rumen microbiota of yaks showed significant differences compared to that of cattle. This adaptive shift in microbial structure also occurs in response to other factors, including seasonal forage changes [76, 77], different feed types [78, 79], and high-grain diet structures [80]. Among the changes observed in the rumen metabolome of yaks, the most prominent were the elevated levels of long-chain fatty acids and nucleotide content. Increased levels of long-chain fatty acids in the rumen may indicate their enhanced microbial synthesis [81–84], while the increased level of nucleotides, particularly cAMP, may reflect adjustments in carbon metabolism [85–88]. These signaling molecules may originate from microbial regulation or host adaptation, highlighting the need for further research on rumen microbiota modulation. Furthermore, functional annotation of rumen microbes revealed enrichment in amino acid degradation and fatty acid metabolism pathways, which may underlie the observed metabolomic changes. Annotation of species associated with significantly differential genes revealed that they were primarily from the genera *Ruminococcus*, *Oscillibacter*, *Selenomonas*, *Schwartzia*, and *Fibrobacter*. Spearman correlation analysis also revealed a positive association between *Ruminococcus* and long-chain fatty acid synthesis. This is also consistent with the findings of Allison et al. [89], who reported the biosynthesis of higher branched-chain fatty acids and aldehydes by members of the phylum Bacillota and Bacteroidota. It was hypothesized that interactions among these microbes may lead to the increased level of long-chain fatty acids biosynthesis in the rumen. Thus, key microorganisms such as *Selenomonas* and *Schwartzia*, which are involved in other metabolic pathways contributing to acetyl CoA production, deserve further attention. *Selenomonas* plays a central role in multiple carbohydrate metabolic pathways, with its metabolic processes, such as acetyl-CoA synthesis, propionate production, and purine metabolism being synergistically driven by related functional microbiota [90]. Analysis of the microbial metabolic network revealed that protozoa may play a major role in signal regulation, with the fatty acid synthesis functions of protozoa and fungi. However, in fatty acid degradation, fungi and protozoa are significantly different in their functions or even negatively correlated, which may indirectly suggest their potential role in regulating the rumen adaptation of yaks. Research on *Schwartzia* is limited; however, it was speculated that it may perform functions similar to *Selenomonas* or provide substrates for gluconeogenesis. Currently, little is known about its in vivo functional roles in the rumen, so this speculation remains to be tested in future studies. Integrating our functional and pathway results [91], our correlative findings suggest that these microbial species, along with auxiliary microbes, may contribute to long-chain fatty acid synthesis. However, due to limited data, further clarification of these microbial interactions is not possible. This necessitates additional research to elucidate the unique adaptive regulatory mechanisms of rumen microbiota of yak. It is important to note that the current study is cross-sectional in nature, which prevents us from establishing causal relationships between the observed microbial shifts and high-altitude adaptation. Longitudinal studies tracking individual animals across altitudinal transitions would be required to confirm these adaptive dynamics.

Secondly, the results of blood metabolomic analysis showed significant changes in pathways of unsaturated fatty acid biosynthesis, primary bile acid biosynthesis, and serine and threonine metabolism in yaks. These correlative results suggest that yaks may rely on fatty acid mobilization and amino acid metabolism to adapt to environmental changes at high altitudes [92, 93]. In contrast, cattle did not exhibit significant shifts in these fatty acid and bile acid metabolic pathways under the same high-altitude conditions. Serum immune indicators revealed that yaks had a significantly increased ALT than in cattle. Metabolic enzymes such as ALP, ALT, and AST in serum are commonly used as non-specific indicators to assess hepatocellular injury or altered liver function, rather than specific markers for particular liver diseases [94–96]. The significantly increased ALT in yak serum may suggest increased hepatic cellular stress or potential liver injury under the given conditions. However, it should be emphasized that serum enzyme activities alone are insufficient to distinguish between pathological liver damage and a controlled physiological stress response. In the absence of direct histological or molecular evidence, this observation should be interpreted with caution. Thus, while the elevated ALT may reflect a greater hepatic burden associated with energy mobilization in yaks, it cannot be conclusively interpreted as either liver pathology or an adaptive mechanism to high-altitude environments [97]. Rumen microorganisms continue to present challenges due to their complex microbial diversity, limited comprehensive genomic data, and difficulty of culturing many species, making it hard to obtain precise biological information through metagenomic approaches. The lack of taxonomic information also hinders the discovery of many microorganisms that are yet to be characterized. In our species annotation, approximately 50% of the microorganisms could not be identified to specific microbial taxa, leading us to suspect the potential influence of unknown species. Furthermore, the current study only included samples from a single region in Qinghai Province. Thus, these findings cannot be generalized for other yak populations across the Qinghai-Tibetan Plateau, as regional differences in breed, diet, and environment may lead to distinct microbial adaptive patterns. With advancements in single-cell sequencing technology, we may be able to directly obtain the full genomes of unculturable rumen microorganisms without relying on cultivation methods. This will provide a wealth of new genetic information for bioinformatics to explore, further enhancing our understanding of this distinct microbial system in the rumen.

In summary, our results highlight the specialized adaptive functions of the yak rumen. However, the specific core functional microorganisms that drive this adaptive process within the rumen microbial community could not be precisely described. This is due to the limited taxonomic information on rumen microorganisms. Future research will need to combine larger-scale multi-regional sampling, in vitro functional validation experiments, and advanced cultivation and sequencing techniques to establish a more comprehensive rumen microbial database, facilitating in-depth mechanistic studies of yak rumen adaptation. This will allow us to explore the complex interactions between host genetics, microbial adaptation, and environmental factors, thereby providing a more comprehensive understanding of high-altitude adaptation in yaks.

## Conclusion

This study provides insights into the specialized adaptation of the yak rumen microbiota to high-altitude environments, based on our regional samples from Qinghai Province. The yak rumen microbiota exhibits a distinct restructuring of microbial communities, characterized by a significant metabolic shift toward the biosynthesis of long-chain fatty acids. This functional reorganization represents a crucial adaptive strategy for surviving in hypoxic, high-altitude conditions. While this microbial adaptation may facilitates survival under hypoxia, it appears to impose physiological costs on the host as observed from microbial shifts and hepatic stress markers. The metabolic shift correlates with a trade-off, potentially contributing to physiological burdens such as elevated hepatic cellular stress, highlighting the delicate balance between adaptation and host health. This study underscores the limitations of current metagenomic approaches, as nearly 50% of the microbial species remained unclassified due to high diversity and limited taxonomic resolution. These findings highlight the need for future research on high-altitude ruminants to integrate advanced techniques such as single-cell sequencing and culture-independent methods. Expanding the rumen microbial genome database is essential to fully decipher the specific microbial drivers of host adaptation and to comprehensively understand the mechanisms by which the microbiota supports survival under high altitudes.

## Electronic Supplementary Material

Below is the link to the electronic supplementary material.

Supplementary Material 1

Supplementary Material 2

## Data Availability

The whole‑metagenome sequencing reads generated in this study have been deposited in the NCBI Sequence Read Archive (SRA) under accession [[PRJNA1296762] (https://www.ncbi.nlm.nih.gov/bioproject/?term=PRJNA1296762)]. The 16 S rRNA gene amplicon sequencing data for yak and cattle rumen samples are available in the SRA under accession [[PRJNA1302440] (https://www.ncbi.nlm.nih.gov/bioproject/?term=PRJNA1302440)].

## Abbreviations

MCP: Microbial Crude Protein
DO: Dissolved Oxygen
AST: Aspartate Aminotransferase
ALT: Alanine Aminotransferase
TP: Total Protein
ALB: Albumin
GLOB: Globulin
GLU: Blood Glucose
TCHO: Total Cholesterol
TRIG: Triglyceride
ALP: Alkaline Phosphatase
TBIL: Total Bilirubin
CK: Creatine Kinase
LDH: Lactate Dehydrogenase
UREA: Urea
UA: Uric Acid
SCFAs: Short-Chain Fatty Acids (acetate, propionate, isobutyrate, butyrate, isovalerate, valerate)
TCD: Thermal Conductivity Detector
CTAB: Cetyltrimethylammonium Bromide
qPCR: Real-Time Quantitative Polymerase Chain Reaction
VIP: Variable in Projection
FDR: False Discovery Rate
SRT: Sunrise Time
SST: Sunset Time
DLD: Daylight Duration
UV: Ultraviolet
MaxT: Maximum Temperature
DRT: Daily Temperature Range
MinT: Minimum Temperature
API: Air Pollution Index
PM10: Particulate Matter ≤ 10 μm
PM2.5: Particulate Matter ≤ 2.5 μm
NO2: Nitrogen Dioxide
SO2: Sulfur Dioxide
CO: Carbon Monoxide
WDO: Water Dissolved Oxygen
WT: Water Temperature
WpH: Water pH
AH: Air Humidity
O3: Ozone
BT: Barn Temperature
BH: Barn Humidity
FI: Food Intake
MBW: Metabolic Body Weight
RR: Respiratory Rate
OMD: Organic Matter Digestibility
DMD: Dry Matter Digestibility
LD: Lignocellulose Digestibility
CPD: Crude Protein Digestibility
ADFD: Apparent Dry Matter Fermentability
TVFA: Total Volatile Fatty Acids
IL: Interleukin
MDA: Malondialdehyde
OGA: Oligosaccharide
IGM: Immunoglobulin M
TNF: Tumor Necrosis Factor

## References

[1] Qiu J, China. The third pole. Nature [Internet]. 2008;454:393–6. 10.1038/454393a. 18650887 10.1038/454393a

[2] Shi P, Chen Y, Zhang G, Tang H, Chen Z, Yu D, et al. Factors contributing to spatial–temporal variations of observed oxygen concentration over the Qinghai-Tibetan Plateau. Sci Rep [Internet]. 2021. 10.1038/s41598-021-96741-6. 11. 34462465 10.1038/s41598-021-96741-6 PMC8405649

[3] Lenfant C. High altitude adaptation in mammals. Am Zool [Internet]. 1973;13:447–56. 10.1093/icb/13.2.447.

[4] Brent MB. A review of the skeletal effects of exposure to high altitude and potential mechanisms for hypobaric hypoxia-induced bone loss. Bone [Internet]. 2022;154:116258. 10.1016/j.bone.2021.116258. 34781048 10.1016/j.bone.2021.116258

[5] Tripathy V, Gupta R. Birth weight among Tibetans at different altitudes in India: Are Tibetans better protected from IUGR? Am J Hum Biol [Internet]. 2005;17:442–50. 10.1002/ajhb.20400. 15981183 10.1002/ajhb.20400

[6] Chun H, Yue Y, Wang Y, Dawa Z, Zhen P, La Q, et al. High prevalence of congenital heart disease at high altitudes in Tibet. Eur J Prev Cardiol [Internet]. 2018;26:756–9. 10.1177/2047487318812502. 30419180 10.1177/2047487318812502

[7] Li J-J, Liu Y, Xie S-Y, Zhao G-D, Dai T, Chen H, et al. Newborn screening for congenital heart disease using echocardiography and follow-up at high altitude in China. Int J Cardiol [Internet]. 2019;274:106–12. 10.1016/j.ijcard.2018.08.102. 30195837 10.1016/j.ijcard.2018.08.102

[8] Rodway GW, Hoffman LA, Sanders MH. High-altitude-related disorders—part I: pathophysiology, differential diagnosis, and treatment. Heart Lung [Internet]. 2003;32:353–9. 10.1016/j.hrtlng.2003.08.002. 14652526 10.1016/j.hrtlng.2003.08.002

[9] Palmer BF, Clegg DJ. Oxygen sensing and metabolic homeostasis. Mol Cell Endocrinol [Internet]. 2014;397:51–8. 10.1016/j.mce.2014.08.001. 25132648 10.1016/j.mce.2014.08.001

[10] Friedman N, Shriker E, Gold B, Durman T, Zarecki R, Ruppin E, et al. Diet-induced changes of redox potential underlie compositional shifts in the rumen archaeal community. Environ Microbiol [Internet]. 2016;19:174–84. 10.1111/1462-2920.13551. 27696646 10.1111/1462-2920.13551

[11] Fenchel T, Finlay B. Oxygen and the Spatial Structure of Microbial Communities. Biol Rev [Internet]. 2008;83:553–69. 10.1111/j.1469-185x.2008.00054.x. 18823390 10.1111/j.1469-185X.2008.00054.x

[12] Clanton TL, Hogan MC, Gladden LB. Regulation of Cellular Gas Exchange, Oxygen Sensing, and Metabolic Control. Compr Physiol [Internet]. 2013;3:1135–90. 10.1002/cphy.c120030. 23897683 10.1002/cphy.c120030

[13] Zhang Z, Xu D, Wang L, Hao J, Wang J, Zhou X, et al. Convergent Evolution of Rumen Microbiomes in High-Altitude Mammals. Curr Biol [Internet]. 2016;26:1873–9. 10.1016/j.cub.2016.05.012. 27321997 10.1016/j.cub.2016.05.012

[14] Pan C, Li H, Mustafa SB, Renqing C, Zhang Z, Li J, et al. Coping with extremes: the rumen transcriptome and microbiome co-regulate plateau adaptability of Xizang goat. BMC Genomics [Internet]. 2024;25. 10.1186/s12864-024-10175-8. 10.1186/s12864-024-10175-8 PMC10921577 38454325

[15] Moraïs S, Mizrahi I. The Road Not Taken: The Rumen Microbiome, Functional Groups, and Community States. Trends Microbiol [Internet]. 2019;27:538–49. 10.1016/j.tim.2018.12.011. 30679075 10.1016/j.tim.2018.12.011

[16] Cammack KM, Austin KJ, Lamberson WR, Conant GC, Cunningham HC. Tiny but mighty: The role of the rumen microbes in livestock production. J Anim Sci [Internet]. 2018;96(2):752–70. 10.1093/jas/skx053. 29385535 10.1093/jas/skx053 PMC6140983

[17] Lin L, Ma H, Zhang J, Yang H, Zhang J, Lai Z, et al. Lignocellulolytic microbiomes orchestrating degradation cascades in the rumen of dairy cattle and their diet-influenced key degradation phases. Anim Adv [Internet]. 2024;1:0–0. 10.48130/animadv-0024-0002.

[18] Rooks MG, Garrett WS. Gut microbiota, metabolites and host immunity. Nat Rev Immunol [Internet]. 2016;16:341–52. 10.1038/nri.2016.42. 27231050 10.1038/nri.2016.42 PMC5541232

[19] Liu K, Zhang Y, Yu Z, Xu Q, Zheng N, Zhao S, et al. Ruminal microbiota–host interaction and its effect on nutrient metabolism. Anim Nutr [Internet]. 2021;7:49–55. 10.1016/j.aninu.2020.12.001. 33997331 10.1016/j.aninu.2020.12.001 PMC8110878

[20] Qiu S, Zhang W, Yu Y, Shi S, Gao Y, Zhang T, et al. Non-pasteurized milk enhances early colonization and persistence of *Lactobacillus* species in dairy calves promoting a stable hindgut microbiome. Anim Adv [Internet]. 2025;2:0–0. 10.48130/animadv-0025-0026.

[21] Alnadari F, Ibeogu IH, Shoura HE, Wang R, Yar MS, Chen C, et al. Immunomodulatory potential of bovine colostrum: a holistic perspective on health, disease resistance, and aging. Anim Adv [Internet]. 2025;2:0–0. 10.48130/animadv-0025-0001.

[22] Gao X, Wang S, Wang Y-F, Li S, Wu S-X, Yan R-G, et al. Long read genome assemblies complemented by single cell RNA-sequencing reveal genetic and cellular mechanisms underlying the adaptive evolution of yak. Nat Commun [Internet]. 2022;13. 10.1038/s41467-022-32164-9. 10.1038/s41467-022-32164-9 PMC9448747 36068211

[23] Kamal M, Cao Y, Gao J, Li N, Zhao X, Chen L, et al. Effects of feeding *Melilotus albus* on growth performance, carcass traits, and meat quality of Hu sheep. Anim Adv [Internet]. 2025;2:0–0. 10.48130/animadv-0025-0008.

[24] Cao D, Wang Z, Saeed HA, Li P, Guo M, Yan J, et al. Handmade cloning: simplicity meets efficiency in modern livestock reproduction. Anim Adv [Internet]. 2025;2:0–0. 10.48130/animadv-0025-0045.

[25] Qinghai Provincial Department of Agriculture and Rural Affairs. Statistical data on meat production by county of animal husbandry in Qinghai Province (2008–2018) [Internet]. National Tibetan Plateau Data Center. 2021. https://data.tpdc.ac.cn/zh-hans/data/644bc4e0-708a-4bcb-b6fb-02460a15d7c2.

[26] Agricultural and Rural Affairs Department of Qinghai Province. Statistical data of livestock production in Qinghai Province by county in the same year (2008–2018) [Internet]. National Tibetan Plateau Data Center. 2021. https://data.tpdc.ac.cn/zh-hans/data/ced0cc9b-3f4d-4b85-ae1e-c7b79bf0688f.

[27] Agricultural and Rural Affairs Department of Qinghai Province. Statistics of livestock production by county in Qinghai Province at the end of the period (2008–2018) [Internet]. National Tibetan Plateau Data Center. 2021. https://data.tpdc.ac.cn/zh-hans/data/81819ee3-a91c-45c0-bf20-e40e8a7fbc49.

[28] Hu X, Chen Y, Huo W, Jia W, Ma H, Ma W, et al. Surface oxygen concentration on the Qinghai-Tibet Plateau (2017–2022). Sci Data [Internet]. 2023. 10.1038/s41597-023-02768-x. 10. 38102189 10.1038/s41597-023-02768-x PMC10724145

[29] AOAC International. Official methods of analysis of AOAC International. Gaithersburg (MD): AOAC International. 1995;1:31–65.

[30] Van Soest PJ, Robertson JB, Lewis BA. Methods for Dietary Fiber, Neutral Detergent Fiber, and Nonstarch Polysaccharides in Relation to Animal Nutrition. J Dairy Sci [Internet]. 1991;74:3583–97. 10.3168/jds.s0022-0302(91)78551-2. 1660498 10.3168/jds.S0022-0302(91)78551-2

[31] Niu D, Zuo S, Jiang D, Tian P, Zheng M, Xu C. Treatment using white rot fungi changed the chemical composition of wheat straw and enhanced digestion by rumen microbiota in vitro. Anim Feed Sci Technol [Internet]. 2018;237:46–54. 10.1016/j.anifeedsci.2018.01.005.

[32] Li Y, Jin W, Cheng Y, Zhu W. Effect of the Associated Methanogen Methanobrevibacter thaueri on the Dynamic Profile of End and Intermediate Metabolites of Anaerobic Fungus Piromyces sp. F1. Curr Microbiol [Internet]. 2016;73:434–41. 10.1007/s00284-016-1078-9. 27287262 10.1007/s00284-016-1078-9

[33] Shen J, Liu Z, Yu Z, Zhu W. Monensin and nisin affect rumen fermentation and microbiota differently in vitro. Front Microbiol [Internet]. 2017;8. 10.3389/fmicb.2017.01111. 10.3389/fmicb.2017.01111 PMC5472720 28670304

[34] Makkar HPS, Sharma OP, Dawra RK, Negi SS. Simple Determination of Microbial Protein in Rumen Liquor. J Dairy Sci [Internet]. 1982;65:2170–3. 10.3168/jds.s0022-0302(82)82477-6. 7153399 10.3168/jds.S0022-0302(82)82477-6

[35] Bradford MM. A rapid and sensitive method for the quantitation of microgram quantities of protein utilizing the principle of protein-dye binding. Anal Biochem [Internet]. 1976;72:248–54. 10.1016/0003-2697(76)90527-3. 942051 10.1016/0003-2697(76)90527-3

[36] Dai Z-L, Zhang J, Wu G, Zhu W-Y. Utilization of amino acids by bacteria from the pig small intestine. Amino Acids [Internet]. 2010;39:1201–15. 10.1007/s00726-010-0556-9. 20300787 10.1007/s00726-010-0556-9

[37] Martin M. Cutadapt removes adapter sequences from high-throughput sequencing reads. EMBnet j [Internet]. 2011;17:10. 10.14806/ej.17.1.200.

[38] Magoč T, Salzberg SL. FLASH: fast length adjustment of short reads to improve genome assemblies. Bioinformatics [Internet]. 2011;27:2957–63. 10.1093/bioinformatics/btr507. 21903629 10.1093/bioinformatics/btr507 PMC3198573

[39] Bolyen E, Rideout JR, Dillon MR, Bokulich NA, Abnet CC, Al-Ghalith GA, et al. Reproducible, interactive, scalable and extensible microbiome data science using QIIME 2. Nat Biotechnol [Internet]. 2019;37:852–7. 10.1038/s41587-019-0209-9. 31341288 10.1038/s41587-019-0209-9 PMC7015180

[40] Bolger AM, Lohse M, Usadel B. Trimmomatic: a flexible trimmer for Illumina sequence data. Bioinformatics [Internet]. 2014;30:2114–20. 10.1093/bioinformatics/btu170. 24695404 10.1093/bioinformatics/btu170 PMC4103590

[41] Li H, Durbin R. Fast and accurate long-read alignment with Burrows–Wheeler transform. Bioinformatics [Internet]. 2010;26:589–95. 10.1093/bioinformatics/btp698. 20080505 10.1093/bioinformatics/btp698 PMC2828108

[42] Danecek P, Bonfield JK, Liddle J, Marshall J, Ohan V, Pollard MO, et al. Twelve years of SAMtools and BCFtools. GigaScience [Internet]. 2021;10. 10.1093/gigascience/giab008. 10.1093/gigascience/giab008 PMC7931819 33590861

[43] Li D, Liu C-M, Luo R, Sadakane K, Lam T-W. MEGAHIT: an ultra-fast single-node solution for large and complex metagenomics assembly via succinct de Bruijn graph. Bioinformatics [Internet]. 2015;31:1674–6. 10.1093/bioinformatics/btv033. 10.1093/bioinformatics/btv033 25609793

[44] Cantalapiedra CP, Hernández-Plaza A, Letunic I, Bork P, Huerta-Cepas J. eggNOG-mapper v2: Functional Annotation, Orthology Assignments, and Domain Prediction at the Metagenomic Scale. Mol Biol Evol [Internet]. 2021;38:5825–9. 10.1093/molbev/msab293. 34597405 10.1093/molbev/msab293 PMC8662613

[45] Buchfink B, Reuter K, Drost H-G. Sensitive protein alignments at tree-of-life scale using DIAMOND. Nat Methods [Internet]. 2021;18:366–8. 10.1038/s41592-021-01101-x. 33828273 10.1038/s41592-021-01101-x PMC8026399

[46] Mirdita M, Steinegger M, Söding J. MMseqs2 desktop and local web server app for fast, interactive sequence searches. Hancock J, editor. Bioinformatics [Internet]. 2019;35:2856–8. 10.1093/bioinformatics/bty1057. 10.1093/bioinformatics/bty1057 PMC6691333 30615063

[47] Li Z, Wang X, Zhang Y, Yu Z, Zhang T, Dai X, et al. Genomic insights into the phylogeny and biomass-degrading enzymes of rumen ciliates. ISME J [Internet]. 2022;16:2775–87. 10.1038/s41396-022-01306-8. 35986094 10.1038/s41396-022-01306-8 PMC9666518

[48] Yan M, Yu Z. The rumen virome database (RVD) [Internet]. Zenodo. 2022. 10.5281/ZENODO.7412085.

[49] Pratt CJ, Chandler EE, Youssef NH, Elshahed MS. Testudinimyces gracilis gen. nov, sp. nov. and Astrotestudinimyces divisus gen. nov, sp. nov., two novel, deep-branching anaerobic gut fungal genera from tortoise faeces. Int J Syst Evol Microbiol [Internet]. 2023;73. 10.1099/ijsem.0.005921. 10.1099/ijsem.0.005921 37252853

[50] Haitjema CH, Gilmore SP, Henske JK, Solomon KV, de Groot R, Kuo A, et al. A parts list for fungal cellulosomes revealed by comparative genomics. Nat Microbiol [Internet]. 2017. 10.1038/nmicrobiol.2017.87. 2. 28555641 10.1038/nmicrobiol.2017.87

[51] Brown JL, Swift CL, Mondo SJ, Seppala S, Salamov A, Singan V, et al. Co–cultivation of the anaerobic fungus Caecomyces churrovis with Methanobacterium bryantii enhances transcription of carbohydrate binding modules, dockerins, and pyruvate formate lyases on specific substrates. Biotechnol Biofuels [Internet]. 2021;14. 10.1186/s13068-021-02083-w. 10.1186/s13068-021-02083-w PMC8665504 34893091

[52] Mondo SJ, He G, Sharma A, Ciobanu D, Riley R, Andreopoulos WB, et al. Consecutive low-frequency shifts in A/T content denote nucleosome positions across microeukaryotes. iScience [Internet]. 2025;28:112472. 10.1016/j.isci.2025.112472. 40491964 10.1016/j.isci.2025.112472 PMC12146615

[53] Wilken SE, Monk JM, Leggieri PA, Lawson CE, Lankiewicz TS, Seppälä S, et al. Experimentally validated reconstruction and analysis of a genome-scale metabolic model of an anaerobic neocallimastigomycota fungus. Lindemann SR, editor. mSystems [Internet]. 2021;6. 10.1128/msystems.00002-21. 10.1128/mSystems.00002-21 PMC8561657 33594000

[54] Youssef NH, Couger MB, Struchtemeyer CG, Liggenstoffer AS, Prade RA, Najar FZ, et al. The Genome of the Anaerobic Fungus Orpinomyces sp. Strain C1A Reveals the Unique Evolutionary History of a Remarkable Plant Biomass Degrader. Appl Environ Microbiol [Internet]. 2013;79:4620–34. 10.1128/aem.00821-13. 23709508 10.1128/AEM.00821-13 PMC3719515

[55] Li Y, Li Y, Jin W, Sharpton TJ, Mackie RI, Cann I, et al. Combined genomic, transcriptomic, proteomic, and physiological characterization of the growth of Pecoramyces sp. F1 in monoculture and co-culture with a syntrophic methanogen. Front Microbiol [Internet]. 2019;10. 10.3389/fmicb.2019.00435. 10.3389/fmicb.2019.00435 PMC6414434 30894845

[56] Xie F, Jin W, Si H, Yuan Y, Tao Y, Liu J, et al. An integrated gene catalog and over 10,000 metagenome-assembled genomes from the gastrointestinal microbiome of ruminants. Microbiome [Internet]. 2021. 10.1186/s40168-021-01078-x. 9. 34118976 10.1186/s40168-021-01078-x PMC8199421

[57] Wood DE, Lu J, Langmead B. Improved metagenomic analysis with Kraken 2. Genome Biol [Internet]. 2019. 10.1186/s13059-019-1891-0. 20. 31779668 10.1186/s13059-019-1891-0 PMC6883579

[58] Cox J, Williams S, Grove K, Lane RH, Aagaard-Tillery KM. A maternal high-fat diet is accompanied by alterations in the fetal primate metabolome. Am J Obstet Gynecol [Internet]. 2009;201:281.e1-281.e9. 10.1016/j.ajog.2009.06.041. 10.1016/j.ajog.2009.06.041 PMC2749563 19733280

[59] R Core Team. R: A language and environment for statistical computing [Internet]. Vienna, Austria: R Foundation for Statistical Computing; 2024. https://www.R-project.org/.

[60] Yu G, Smith D, Zhu H, Guan Y, Lam TT-Y, ggtree. An R package for visualization and annotation of phylogenetic trees with their covariates and other associated data. Methods Ecol Evol [Internet]. 2016;8:28–36. 10.1111/2041-210X.12628.

[61] Xu S, Dai Z, Guo P, Fu X, Liu S, Zhou L, et al. ggtreeExtra: compact visualization of richly annotated phylogenetic data. Tamura K, editor. Mol Biol Evol [Internet]. 2021;38:4039–42. 10.1093/molbev/msab166. 10.1093/molbev/msab166 PMC8382893 34097064

[62] Wickham H. ggplot2: elegant graphics for data analysis [Internet]. Springer-Verlag New York. 2016. https://ggplot2.tidyverse.org.

[63] Wickham H, Averick M, Bryan J, Chang W, McGowan L, François R, et al. Welcome to the Tidyverse. J Open Source Softw [Internet]. 2019;4:1686. 10.21105/joss.01686.

[64] Yu G. Data integration, manipulation and visualization of phylogenetic trees [Internet]. 2022. 10.1201/9781003279242.

[65] Wickham H, François R, Henry L, Müller K, Vaughan D. dplyr: a grammar of data manipulation [Internet]. 2023. https://dplyr.tidyverse.org.

[66] Wickham H. Reshaping data with the reshape package. J Stat Softw [Internet]. 2007;21(12). 10.18637/jss.v021.i12.

[67] Campitelli E. ggnewscale: multiple fill and colour scales in ggplot2 [Internet]. CRAN: Contributed Packages. The R Foundation. 2019. 10.32614/cran.package.ggnewscale.

[68] Garnier S, Ross N, BoB Rudis, Filipovic-Pierucci A, Galili T, Timelyportfolio, et al. sjmgarnier/viridis: CRAN release v0.6.3 [Internet]. 2023. 10.5281/ZENODO.4679423.

[69] ggpubr KA. ggplot2 based publication ready plots [Internet]. CRAN: Contributed Packages. The R Foundation; 2016. 10.32614/cran.package.ggpubr.

[70] Couture-Beil A. rjson: JSON for R [Internet]. CRAN: Contributed Packages. The R Foundation. 2007. 10.32614/cran.package.rjson.

[71] McKinney W. Data structures for statistical computing in Python. Proceedings of the Python in Science Conference [Internet]. 2010. pp. 56–61. 10.25080/majora-92bf1922-00a.

[72] Slowikowski K. ggrepel: automatically position non-overlapping text labels with ggplot2 [Internet]. CRAN: Contributed Packages. The R Foundation. 2016. 10.32614/cran.package.ggrepel.

[73] Huerta-Cepas J, Serra F, Bork P. ETE 3: Reconstruction, Analysis, and Visualization of Phylogenomic Data. Mol Biol Evol [Internet] (OUP). 2016;33:1635–8. 10.1093/molbev/msw046. 10.1093/molbev/msw046 PMC4868116 26921390

[74] Virtanen P, Gommers R, Oliphant TE, Haberland M, Reddy T, Cournapeau D, et al. SciPy 1.0: fundamental algorithms for scientific computing in Python. Nat Methods [Internet]. 2020;17:261–72. 10.1038/s41592-019-0686-2. 32015543 10.1038/s41592-019-0686-2 PMC7056644

[75] The pandas development team. pandas-dev/pandas: Pandas [Internet]. Zenodo; 2024. 10.5281/ZENODO.3509134.

[76] Ma L, Xu S, Liu H, Xu T, Hu L, Zhao N, et al. Yak rumen microbial diversity at different forage growth stages of an alpine meadow on the Qinghai-Tibet Plateau. PeerJ [Internet]. 2019;7:e7645. 10.7717/peerj.7645. 31579584 10.7717/peerj.7645 PMC6754979

[77] Guo N, Wu Q, Shi F, Niu J, Zhang T, Degen AA, et al. Seasonal dynamics of diet–gut microbiota interaction in adaptation of yaks to life at high altitude. npj Biofilms Microbiomes [Internet]. 2021. 10.1038/s41522-021-00207-6. 7. 33879801 10.1038/s41522-021-00207-6 PMC8058075

[78] Lengowski MB, Zuber KHR, Witzig M, Möhring J, Boguhn J, Rodehutscord M. Changes in Rumen Microbial Community Composition during Adaption to an In Vitro System and the Impact of Different Forages. Virolle M-J. editor PLoS ONE [Internet]. 2016;11:e0150115. 10.1371/journal.pone.0150115. 10.1371/journal.pone.0150115 PMC4771158 26928330

[79] de Menezes AB, Lewis E, O’Donovan M, O’Neill BF, Clipson N, Doyle EM. Microbiome analysis of dairy cows fed pasture or total mixed ration diets. FEMS Microbiol Ecol [Internet]. 2011;78:256–65. 10.1111/j.1574-6941.2011.01151.x. 21671962 10.1111/j.1574-6941.2011.01151.x

[80] Fernando SC, Purvis HT II, Najar FZ, Sukharnikov LO, Krehbiel CR, Nagaraja TG, et al. Rumen Microbial Population Dynamics during Adaptation to a High-Grain Diet. Appl Environ Microbiol [Internet]. 2010;76:7482–90. 10.1128/aem.00388-10. 20851965 10.1128/AEM.00388-10 PMC2976194

[81] Wu Z, Palmquist DL. Synthesis and Biohydrogenation of Fatty Acids by Ruminal Microorganisms In Vitro. J Dairy Sci [Internet]. 1991;74:3035–46. 10.3168/jds.s0022-0302(91)78489-0. 1779057 10.3168/jds.S0022-0302(91)78489-0

[82] Wu Z, Ohajuruka OA, Palmquist DL. Ruminal Synthesis, Biohydrogenation, and Digestibility of Fatty Acids by Dairy Cows. J Dairy Sci [Internet]. 1991;74:3025–34. 10.3168/jds.s0022-0302(91)78488-9. 1779056 10.3168/jds.S0022-0302(91)78488-9

[83] Chalupa W, Rickabaugh B, Kronfeld D, David Sklan S. Rumen Fermentation In Vitro as Influenced by Long Chain Fatty Acids. J Dairy Sci [Internet]. 1984;67:1439–44. 10.3168/jds.s0022-0302(84)81459-9. 6747049 10.3168/jds.s0022-0302(84)81459-9

[84] Doreau M, Ferlay A. Digestion and utilisation of fatty acids by ruminants. Anim Feed Sci Technol [Internet]. 1994;45:379–96. 10.1016/0377-8401(94)90039-6.

[85] Kalia D, Merey G, Nakayama S, Zheng Y, Zhou J, Luo Y, et al. Nucleotide, c-di-GMP, c-di-AMP, cGMP, cAMP, (p)ppGpp signaling in bacteria and implications in pathogenesis. Chem Soc Rev [Internet]. 2013;42:305–41. 10.1039/c2cs35206k. 23023210 10.1039/c2cs35206k

[86] Rabinowitz JD, Silhavy TJ. Metabolite turns master regulator. Nature [Internet]. 2013;500:283–4. 10.1038/nature12544. 23925111 10.1038/nature12544 PMC4084598

[87] You C, Okano H, Hui S, Zhang Z, Kim M, Gunderson CW, et al. Coordination of bacterial proteome with metabolism by cyclic AMP signalling. Nature [Internet]. 2013;500:301–6. 10.1038/nature12446. 23925119 10.1038/nature12446 PMC4038431

[88] Shimada T, Fujita N, Yamamoto K, Ishihama A. Novel roles of cAMP Receptor protein (CRP) in regulation of transport and metabolism of carbon sources. Herman C, editor. PLoS ONE [Internet]. 2011;6:e20081. 10.1371/journal.pone.0020081. 10.1371/journal.pone.0020081 PMC3105977 21673794

[89] Allison MJ, Bryant MP, Katz I, Keeney M. Metabolic function of branched-chain volatile fatty acids, growth factors for ruminococci II. J Bacteriol [Internet]. 1962;83:1084–93. 10.1128/jb.83.5.1084-1093.1962. 13860622 10.1128/jb.83.5.1084-1093.1962 PMC279411

[90] Xue M, Xie Y, Zang X, Zhong Y, Ma X, Sun H, et al. Deciphering functional groups of rumen microbiome and their underlying potentially causal relationships in shaping host traits. iMeta [Internet]. 2024. 10.1002/imt2.225. 3. 39135684 10.1002/imt2.225 PMC11316931

[91] Ricke SC, Martin SA, Nisbet DJ. Ecology, Metabolism, and Genetics of Ruminal Selenomonads. Crit Rev Microbiol [Internet]. 1996;22:27–65. 10.3109/10408419609106455. 8729959 10.3109/10408419609106455

[92] Zheng J, Du M, Zhang J, Liang Z, Ahmad AA, Shen J, et al. Transcriptomic and metabolomic analyses reveal inhibition of hepatic adipogenesis and fat catabolism in yak for adaptation to forage shortage during cold season. Front Cell Dev Biol [Internet]. 2022;9. 10.3389/fcell.2021.759521. 10.3389/fcell.2021.759521 PMC8802892 35111749

[93] Huang C, Ge F, Yao X, Guo X, Bao P, Ma X, et al. Microbiome and metabolomics reveal the effects of different feeding systems on the growth and ruminal development of yaks. Front Microbiol [Internet]. 2021;12. 10.3389/fmicb.2021.682989. 10.3389/fmicb.2021.682989 PMC8265505 34248900

[94] McGill MR. The past and present of serum aminotransferases and the future of liver injury biomarkers. EXCLI J [Internet]. 2016;15:817–28. 10.17179/EXCLI2016-800. 28337112 10.17179/excli2016-800 PMC5318690

[95] Dufour DR, Lott JA, Nolte FS, Gretch DR, Koff RS, Seeff LB. Diagnosis and Monitoring of Hepatic Injury. II. Recommendations for Use of Laboratory Tests in Screening, Diagnosis, and Monitoring. Clin Chem [Internet]. 2000;46:2050–68. 10.1093/clinchem/46.12.2050. 11106350 10.1093/clinchem/46.12.2050 PMC7110382

[96] Lescot T, Karvellas C, Beaussier M, Magder S, Riou B. Acquired Liver Injury in the Intensive Care Unit. Anesthesiology [Internet]. 2012;117:898–904. 10.1097/aln.0b013e318266c6df. 22854981 10.1097/ALN.0b013e318266c6df

[97] Huang M, Zhang X, Yan W, Liu J, Wang H. Metabolomics reveals potential plateau adaptability by regulating inflammatory response and oxidative stress-related metabolism and energy metabolism pathways in yak. J Anim Sci Technol [Internet]. 2022;64:97–109. 10.5187/jast.2021.e129. 35174345 10.5187/jast.2021.e129 PMC8819316

