# Supplementary material for: The price of survival: comparative adaptation to high altitudes between yaks and cattle
Source: Anim Microbiome. 2026 Jun 6;8:92. doi: 10.1186/s42523-026-00584-3 (PMC13466131; doi:10.1186/s42523-026-00584-3)
Supplement: Supplementary file 1 [file 42523_2026_584_MOESM1_ESM.docx]

**Fig. S1.** 16S rarefaction curve and blood metabolome functional annotation.

**Fig. S2.** Functional Annotation to Species Proportion Workflow.

**Fig. S3.** Environmental and physiological parameters across low- and high-altitude regions.(A) sunrise time(SRT ,HH:MM), sunset time(SST ,HH:MM), and daylight duration(DLD ,hours min), ultraviolet (UV ,index);(B) minimum temperature(MinT ,℃), maximum temperature(MaxT ,℃), diurnal temperature range(DTR ,℃);(C) oxygen (O₂ ,%);(D) air humidity (AH ,%), ozone (O₃ ,µg/m^3^), air pollution index (API ,index), particulate matter (PM10, PM2.5 ,µg/m^3^), nitrogen dioxide (NO₂ ,µg/m^3^), sulfur dioxide (SO₂ ,µg/m^3^), carbon monoxide (CO ,mg/m^3^), The right y-axis shows the CO correspondence information;(E) water dissolved oxygen (WDO ,mg/L), water temperature(WT ,℃), water pH(WpH), The right y-axis shows the WpH correspondence information;(F) barn temperature(BT, ℃), barn humidity(BH ,%). Significant differences between low- and high-altitude regions are denoted as follows: ns p > 0.05 *p < 0.05, **p < 0.01, ***p < 0.001, ****p < 0.0001

**Fig. S4.** Phenotypic indicators:(A) Metabolic Body Weight (MBW, g/kg W^0.75^), Food Intake (FI, g/kg W^0.75^), Water Intake (WI, g/kg W^0.75^) ,The right y-axis shows the WI correspondence information; (B) Heart Rate (HR, beats/min), Respiratory Rate (RR, breaths/min); (C) Organic Matter Digestibility (OMD, %), Dry Matter Digestibility (DMD, %), Neutral Detergent Fiber Digestibility (NDFD, %), Apparent Dry Matter Fermentability (ADFD, %), Lignocellulose Digestibility (LD, %), Crude Protein Digestibility (CPD, %). Significant differences between low- and high-altitude regions are denoted as follows: ns p > 0.05 *p < 0.05, **p < 0.01, ***p < 0.001, ****p < 0.0001

**Fig. S5.** Rumen fermentation parameters:(A) pH, Ammonia Nitrogen (NH_3_-N, mg/dL), Microbial Crude Protein (MCP, mg/dL); (B) Total Volatile Fatty Acids (TVFA, mmol/L); (C) Isobutyric Acid (IBA, mmol/L), Butyric Acid (BA, mmol/L), Propionic Acid (PA, mmol/L), Acetic Acid (AA, mmol/L) ,The right y-axis shows the AA correspondence information. Significant differences between low- and high-altitude regions are denoted as follows: ns p > 0.05, *p < 0.05, **p < 0.01, ***p < 0.001, ****p < 0.0001

**Fig. S6.** Blood Metabolism and Immunity: (A) Alanine Aminotransferase (ALT, U/L), Total Bilirubin (TBIL, µmol/L), Glucose (GLU, mmol/L);(B) Tumor Necrosis Factor (TNF, pg/mL), Interleukin (IL, pg/mL), Malondialdehyde (MDA, nmol/mL), Immunoglobulin A (IGA, µg/L), Immunoglobulin M (IGM, µg/L). For panel B, data were log-transformed (log10) for analysis. Significant differences between low- and high-altitude regions are denoted as follows: ns p > 0.05,*p < 0.05, **p < 0.01, ***p < 0.001, ****p < 0.0001

**Fig. S7.** The slaughter rate and meat yield of yaks vary with oxygen content and Blood Supplementary Indicators. (A) Slaughter rate with annual mean oxygen content;(B) Meat production with annual mean oxygen content. (C) Albumin to Globulin ratio (A/G), Urea (UREA, mmol/L), Gamma-glutamyl transferase (Ga, U/L), Phosphorus (P, mmol/L), Total Cholesterol (TCHO, mmol/L), Triglycerides (TRIG, mmol/L);(D) Aspartate Aminotransferase (AST, U/L), Total Protein (TP, g/L), Albumin (ALB, g/L), Globulin (GLOB, g/L), Alkaline Phosphatase (ALP, U/L), Creatine Kinase (CK, U/L), Lactic Dehydrogenase (LDH, U/L), Uric Acid (UA, µmol/L). Data were log-transformed (log10) for analysis. Significant differences between low- and high-altitude regions are denoted as follows: ns p > 0.05
